# The activation of mGluR4 rescues parallel fiber synaptic transmission and LTP, motor learning and social behavior in a mouse model of Fragile X Syndrome

**DOI:** 10.1186/s13229-023-00547-4

**Published:** 2023-04-07

**Authors:** Ricardo Martín, Alberto Samuel Suárez-Pinilla, Nuria García-Font, M. Luisa Laguna-Luque, Juan C. López-Ramos, María Jesús Oset-Gasque, Agnes Gruart, José M. Delgado-García, Magdalena Torres, José Sánchez-Prieto

**Affiliations:** 1 Departamento de Bioquímica y Biología Molecular, Facultad de Veterinaria, Universidad Complutense, Instituto Universitario de Investigación en Neuroquímica, 28040 Madrid, Spain; 2 Instituto de Investigación Sanitaria del Hospital Clínico San Carlos, 28040 Madrid, Spain; 3 Departamento de Fisiología, Facultad de Medicina, Universidad Complutense, 28040 Madrid, Spain; 4 Centre for Discovery Brain Sciences and Simon Initiative for Developing Brain, University of Edinburgh, Edinburgh, EH89JZ UK; 5 Division de Neurociencias, Universidad Pablo de Olavide, 41013 Sevilla, Spain; 6 Departamento de Bioquímica, Facultad de Farmacia, Universidad Complutense, Instituto Universitario Investigación en Neuroquímica, 28040 Madrid, Spain

**Keywords:** Parallel fiber-Purkinje cell synapse, β adrenergic receptor, RRP size, *Fmr1* KO, Classical conditioning, Vestibuloocular reflex

## Abstract

**Background:**

Fragile X syndrome (FXS), the most common inherited intellectual disability, is caused by the loss of expression of the Fragile X Messenger Ribonucleoprotein (FMRP). FMRP is an RNA-binding protein that negatively regulates the expression of many postsynaptic as well as presynaptic proteins involved in action potential properties, calcium homeostasis and neurotransmitter release. FXS patients and mice lacking FMRP suffer from multiple behavioral alterations, including deficits in motor learning for which there is currently no specific treatment.

**Methods:**

We performed electron microscopy, whole-cell patch-clamp electrophysiology and behavioral experiments to characterise the synaptic mechanisms underlying the motor learning deficits observed in *Fmr1*KO mice and the therapeutic potential of positive allosteric modulator of mGluR4.

**Results:**

We found that enhanced synaptic vesicle docking of cerebellar parallel fiber to Purkinje cell *Fmr1*KO synapses was associated with enhanced asynchronous release, which not only prevents further potentiation, but it also compromises presynaptic parallel fiber long-term potentiation (PF-LTP) mediated by β adrenergic receptors. A reduction in extracellular Ca^2+^ concentration restored the readily releasable pool (RRP) size, basal synaptic transmission, β adrenergic receptor-mediated potentiation, and PF-LTP. Interestingly, VU 0155041, a selective positive allosteric modulator of mGluR4, also restored both the RRP size and PF-LTP in mice of either sex. Moreover, when injected into *Fmr1*KO male mice, VU 0155041 improved motor learning in skilled reaching, classical eyeblink conditioning and vestibuloocular reflex (VOR) tests, as well as the social behavior alterations of these mice.

**Limitations:**

We cannot rule out that the activation of mGluR4s via systemic administration of VU0155041 can also affect other brain regions. Further studies are needed to stablish the effect of a specific activation of mGluR4 in cerebellar granule cells.

**Conclusions:**

Our study shows that an increase in synaptic vesicles, SV, docking may cause the loss of PF-LTP and motor learning and social deficits of *Fmr1*KO mice and that the reversal of these changes by pharmacological activation of mGluR4 may offer therapeutic relief for motor learning and social deficits in FXS.

**Supplementary Information:**

The online version contains supplementary material available at 10.1186/s13229-023-00547-4.

## Background

Fragile X syndrome (FXS), the most common inherited intellectual disability, is associated with cognitive deficits, hyperactivity, anxiety and impaired social interactions [1, 2]. FXS is caused by the silencing of the *Fmr1* gene, which encodes the Fragile X Messenger Ribonucleoprotein (FMRP). FMRP is an RNA-binding protein that negatively regulates protein synthesis [3] and in its absence, the expression of many postsynaptic proteins is altered, affecting long-term forms of postsynaptic plasticity [4]. FMRP can be found in axons and presynaptic nerve terminals [5, 6]. Indeed, proteomic studies on a mouse model of FXS, *Fmr1*KO mice, revealed a presynaptic phenotype [7], with altered expression of presynaptic proteins involved in excitability, Ca^2+^ homeostasis and neurotransmitter release [6, 8–10]. Electron microscopy (EM) studies of *Fmr1*KO synapses identified an increase in the number of docked synaptic vesicles (SVs)[11–13]. Changes in SV docking could affect neurotransmitter release, as the number of docked vesicles is correlated with the size of the readily releasable pool (RRP) of SVs [14, 15]. Specifically, the enhanced SV docking at *Fmr1*KO synapses may prevent presynaptic forms of synaptic plasticity, such as parallel fiber to Purkinje cell Long Term Potentiation (PF-LTP), which is expressed through an increase in neurotransmitter release.

FXS patients also experience deficits in the acquisition of motor skills, affecting fine and gross motor activities [16]. Plasticity at PF-PC synapses is involved in cerebellar motor learning [17–19] and this may be related to the motor learning deficits described in *Fmr1*KO mice [20]. Thus, we hypothesized that rescuing the eventual loss of PF-PC LTP in *Fmr1*KO mice could help ameliorate their motor learning deficits [20].

Here we found that asynchronous release is increased, while PF-PC LTP is lost in *Fmr1*KO synapses because they have more docked SVs in the basal state and a larger RRP size than wild type (WT) synapses, such that β-AR mediated potentiation is prevented. Lowering extracellular Ca^2+^ concentration ([Ca^2+^]_e_) from 2.5 to 1 mM restored these parameters. These ameliorating effects were also produced by the selective positive allosteric modulator (PAM) of mGluR4, VU 015504, which reduces Ca^2+^ influx at nerve terminals. VU 0155041 is active in vivo [21, 22] and has been used in animal models of Parkinson disease [21, 23] and of autistic syndrome [24]. Interestingly, the motor learning of *Fmr1*KO mice that received VU 0155041 improved, as did their social interactions. Thus, pharmacological activation of mGluR4 may restore motor and behavioral deficits in FXS.

## Materials and methods

### Mice

*Fmr1*KO (Strain #:003025, RRID:IMSR_JAX:003025) or WT (Strain #:000664, RRID:IMSR_JAX:000664) mice were used to establish the colonies at the Animal House Service at the Complutense University, an authorized center for the breeding of genetically modified mice. *Fmr1*KO mice and WT littermate were used in this study. These mice were also supplied to the Pablo de Olavide Animal House (Sevilla, Spain). Experiments were carried out in accordance with guidelines of the European Union Council (2010/276:33-79/EU) and Spanish (BOE 34:11370-421, 2013) regulations for the use of laboratory animals in chronic studies and, in addition, were approved by the Ethics Committee of Comunidad de Madrid (PROEX 012/18) and of the Junta de Andalucía (code 06/04/2020/049).

### Synaptosome preparation

*Fmr1*KO mice or WT littermate (3 months old mice of either sex) were anaesthetized with isoflurane (1.5–2% in a mixture of 80% synthetic air/20% oxygen) and sacrificed by decapitation. Synaptosomes were purified from 4–5 cerebella on discontinuous Percoll gradients (GE Healthcare, Uppsala, Sweden). Briefly, the tissue was homogenized and centrifuged for 2 min at 2000×*g*, and the supernatant was then centrifuged again for 12 min at 9500×*g*. The pellets obtained were gently resuspended in 0.32 M sucrose [pH 7.4] and placed onto a 3 ml Percoll discontinuous gradient containing: 0.32 M sucrose; 1 mM EDTA; 0.25 mM DL-dithiothreitol; and 3, 10 or 23% Percoll [pH 7.4]. After centrifugation at 25,000×*g* for 10 min at 4 °C, the synaptosomes were recovered from between the 10% and 23% Percoll bands, and they were resuspended in HEPES buffered medium (HBM) [25]. The preparation of cerebellar synaptosomes largely represents the synaptic boutons of granular cells, by far the most abundant cells in the brain [26].

### Glutamate release

Glutamate release from cerebellar synaptosomes was assayed by on-line fluorimetry [25] based on the reduction of NADP^+^ (1 mM: Calbiochem) by glutamate dehydrogenase (Sigma-Aldrich, St. Louis, MO, USA). The fluorescence of the NADPH generated was measured in a Perkin Elmer LS-50 luminescence spectrometer at excitation and emission wavelengths of 340 and 460 nm, respectively, and using FL WinLab v. 4.00.02 software. Spontaneous glutamate release was determined in the presence of the Na^+^-channel blocker, Tetrodotoxin (TTx, 1 μM: Abcam, Cambridge, UK).

### cAMP accumulation

The accumulation of cAMP was determined using a cAMP dynamic 2 kit (Cisbio, Bioassays, Bagnols sur-Cèze, France). Synaptosomes were incubated for 1 h at 37 °C (0.67 mg/ml) in HBM and after 15 min, 1.33 mM Ca^2+^ and 1 mM of the cAMP phosphodiesterase inhibitor, 3-isobutyl-1-methylxanthine (IBMX: Calbiochem, Damstard, Germany), were added to the synaptosomes for 15 min. Isoproterenol (100 µM) was then added for 10 min, the synaptosomes were collected by centrifugation and transferred to a 96-well assay plate, and the assay components were added having been diluted in lysis buffer: the europium cryptate-labeled anti cAMP antibody and the d2-labeled cAMP analog. After incubation for 1 h at room temperature (RT), the europium cryptate fluorescence and TR-FRET signals were measured over 50 ms on a FluoStar Omega microplate reader (BMG Lab Technologies, Offenburg, Germany) at 620 and 665 nm, respectively, after excitation at 337 nm (see [13]). The data were obtained using Omega BMG Labtech v.1.00 software.

### Immunofluorescence

Immunofluorescence was performed using an affinity-purified rabbit polyclonal antiserum against β_1_-AR (1:200, Cat# sc-568: Santa Cruz Biotechnology, RRID:AB_2225388) and a mouse monoclonal antibody against synaptophysin 1 (1:500, Cat# 101 011: Synaptic Systems, RRID:AB_887822), as described previously [27]. As a control for the immunofluorescence, the primary antibodies were omitted from the staining procedure, whereupon no immunoreactivity resembling that obtained with the specific antibodies was evident. After washing in Tris Buffered Saline, TBS, the labelled synaptosomes were incubated for 2 h with secondary antibodies diluted in Tris TBS: Alexa fluor 488 Donkey anti-mouse IgG (1:500, Cat# A-21202: Invitrogen, RRID:AB_141607) and Alexa fluor 594 Donkey anti-rabbit IgG (1:500, Cat# A-21207: Invitrogen, RRID:AB_141637). After several washes in TBS, the coverslips were mounted in Prolong Antifade Kit (Molecular Probes, Eugene, OR, USA), and the synaptosomes were viewed on a Nikon Diaphot microscope equipped with a 100 × objective, a mercury lamp light source and fluorescein-rhodamine Nikon filter sets. Images were acquired with AQM 4800_80 software and analyzed using Image J 1.43 m software.

### Western blotting

The P2 crude synaptosomal fraction (4 μg of protein per lane) was diluted in Laemmli loading buffer with β-mercaptoethanol (5% v/v), resolved by SDS-PAGE (8% acrylamide, Bio-Rad), and analyzed in Western blots following standard procedures. The proteins were transferred to PVDF membranes (Hybond ECL: GE Healthcare Life Sciences, Madrid, Spain) and after several washes, the membranes were probed with a polyclonal rabbit anti-β1-AR antiserum diluted 1:200 (Santa Cruz Biotechnology Cat# sc-568, RRID:AB_2225388) and a monoclonal mouse anti-β-actin antibody diluted 1:5000 (Sigma cat# A2228, RRID:AB_476697). After several washes, the membranes were incubated with the corresponding IRD-labeled secondary antibodies: goat anti-rabbit and goat anti-mouse coupled to IRDye 800 (LI-COR Biosciences Cat# 925-32211, RRID:AB_2651127) or IRDye 680 (LI-COR Biosciences Cat# 925-68020, RRID:AB_2687826). The membranes were scanned in an Odyssey Infrared imaging system, and the immunolabeling of proteins was compared by densitometry and quantified using Odyssey 2.0 software. The data were normalized to the β-actin signal to account for loading differences.

### Cytosolic free Ca^2+^

The cytosolic free Ca^2+^ concentration ([Ca^2+^]_c_) was measured with fura-2. P2 crude synaptosomes were resuspended in HBM (1.5 mg/ml) with 16 μM BSA in the presence of CaCl_2_ (1.3 mM) and fura-2-acetoxymethyl ester (fura 2-AM, 5 μM: Molecular Probes, Eugene, OR, USA), and they were incubated at 37 °C for 25 min. After fura-2 loading, the synaptosomes were pelleted and resuspended in 1.1 ml fresh HBM medium without BSA. A 1 ml aliquot was transferred to a stirred cuvette and CaCl_2_ (1.3 mM) was added. Fluorescence was monitored at 340 and 510 nm, taking data points at 0.3 s intervals, and the [Ca^2+^]_c_ was calculated using the equations described previously [28]. Synaptosomes were depolarized with a low KCl concentrations (10 mM KCl) to induce synaptic events involving Na^+^, K^+^ and Ca^2+^ channel firing which are compatible with the generation of action potentials [29].

### Electrophysiology

*Fmr1* KO mice or WT littermate (18–30 days old mice of either sex) were anaesthetized with isoflurane (1.5–2% in a mixture of 80% synthetic air/20% oxygen) and sacrifice by decapitation. Cerebellar parasagittal slices (325 µm thick) were obtained in ice-cold Ringer’s solution [119 mM NaCl, 2.5 mM KCl, 1.3 mM MgSO_4_, 2.5 mM CaCl_2_, 26 mM NaHCO_3_, 1 mM NaH_2_PO_4_, 10 mM glucose] on a Leica VT 1200S vibratome. The slices were kept in a holding chamber containing Ringer’s solution for at least 1 h and then transferred to a superfusion chamber for recording. The Ringer’s solution in the superfusion chamber was supplemented with 0.1 mM picrotoxin to block the GABA_A_ receptors and it was bubbled with 95% O_2_/5% CO_2_ at a flow rate of 1 mL/min. Recordings of the PF-PC synapses were obtained at 25 ºC using a temperature controller (TC-324C Warner-Instruments) as described previously [30].

Theta capillaries with a 2–5 µm tip and filled with Ringer’s solution were used for bipolar stimulation. The electrodes were connected to a stimulator (S38, GRASS) through an isolation unit and placed near the pial surface of the molecular layer to stimulate PF input to PCs. Stimuli (< 50 pA, 100 ms) were delivered at 0.05 Hz with paired pulses applied 80 ms apart to obtain the PPR as EPSC_2_/EPSC_1_.

Whole cell recordings from individual PCs were obtained with a PC-ONE amplifier under voltage-clamp conditions and the membrane potential was held at − 70 mV to record glutamatergic evoked EPSCs (eEPSCs). Signals were fed to a Pentium-based PC through a DigiData1322A interface board (Axon Instruments) and the pCLAMP 10.2 software was used to generate stimuli, as well as for data display, acquisition, storage and analysis. Patch pipettes (3–4 MΩ) were pulled from thin-walled borosilicate glass (1.5 mm outer diameter) on a P-97 puller (Sutter-Instrument), and they were filled with an internal solution containing: 122.5 mM cesium gluconate, 10 mM HEPES, 10 mM BAPTA, 2 mM Mg-ATP, 8 mM NaCl and 5 mM QX-314-Br (pH 7.3 adjusted with CsOH, osmolarity 290 mOsm). Series and input resistances were monitored throughout the experiment using a − 5 mV pulse, and the recordings were considered stable when the series and input resistances, resting membrane potential and stimulus artifact duration did not change > 20%. Cells that did not meet these criteria were discarded. To avoid irreversible effects of agonists/antagonists/inhibitors or LTP protocols, only one neuron per slice was analyzed. Recordings were also made in cerebellar slices from *Fmr1* KO mice 2 h after intraperitoneal injection of the mGluR4 PAM, VU 0155041 (5 mg/kg) [31] or the saline vehicle alone.

To measure aEPSCs, CaCl_2_ was replaced with 2.5 mM SrCl_2_, to be sure that the evoked sEPSC is equivalent across slices and mice, the stimuli were adjusted to yield an amplitude between 250 and 500 pA, and they were delivered every 20 s. Asynchronous release associated with each stimulus was estimated after 20 ms and over 500 ms. The asynchronous events associated with the last six stimuli of a 5 min period were quantified in the basal condition and after 10 min in the presence of isoproterenol (100 μM).

For LTP induction, a tetanic train of 100 stimuli delivered at 10 Hz was applied at least 15 min after the beginning of the recording to permit adequate time for the diffusion of BAPTA into the dendritic tree. The baseline PF EPSC amplitude was less than 300 pA to avoid sodium spikes that escaped voltage clamp, particularly during and after tetanization. PF-LTP in WT slices shows a low sensitivity to extracellular Ca^2+^ as it is absent at 1.0 mM [Ca^2+^]_e_ [32], therefore PF-LTP was performed at 2.5 mM [Ca^2+^]_e_, while rescue experiments in *Fmr1* KO slices were performed at 1.0 mM [Ca^2+^]_e_.

The size of the RRP was estimated as described previously [33]. To minimize the variability of these estimates, the stimulus intensity prior to LTP induction was adjusted to yield EPSC amplitudes between 150–200 pA. A tetanic train (100 stimuli at 40 Hz) was applied 30 min after application of the 10 Hz train to induce presynaptic LTP. The cumulative EPSC amplitudes during this train were plotted and the y-intercept that extended from the linear part of the curve (times longer than 1.5 s, when the cumulative amplitude curve reaches a steady state) was used to estimate the size of the RRP. When the effect of decreasing [Ca^2+^]_e_ on RRP size was studied, the slices were maintained at low Ca^2+^ for at least 1 h and they were incubated with 2.5 mM Ca^2+^ for 3 min prior to applying the tetanic train. OriginLab 8 software was used for plotting and fitting.

### Electron microscopy analysis of synaptic vesicle distribution at the active zone

Parasagittal slices (325 µm thick) from the cerebellum were obtained as described above for the electrophysiology experiments, transferred to an immersion recording chamber and superfused at 1 mL min^−1^ with gassed Ringer’s solution including 0.1 mM picrotoxin. In some cases, the slices were also treated with isoproterenol (100 µM) for 10 min. The slices were fixed immediately afterwards by immersion in 3.5% glutaraldehyde in 0.1 M PB (pH 7.4) at 37 °C for 45 min and they were then left in glutaraldehyde solution for 30 min at RT before storing them for 20 h at 4 °C. The slices were then rinsed six times with large volumes of 0.1 M Phosphate Buffer (PB) and post-fixing them in 1% OsO_4_–1.5% K_3_Fe(CN)_6_ for 1 h at RT. After dehydrating through a graded series of ethanol (30, 50, 70, 80, 90, 95 and 100%), the samples were then embedded using the SPURR embedding kit (TAAB, Aldermaston, UK). Ultrathin ultramicrotome sections (70–80 nm thick: Leica EM UC6 Leica Microsystems, Wetzlar, Germany) were routinely stained with uranyl acetate and lead citrate, and images were obtained on a Jeol 1010 transmission electron microscope (Jeol, Tokyo, Japan). Randomly chosen areas of the cerebellar molecular layer were then photographed at 80,000× magnification and only asymmetric synapses with clearly identifiable electron-dense postsynaptic densities were analyzed with ImageJ software. The number of SVs was determined by measuring the distance between the outer layer of the vesicle and the inner layer of the AZ membrane, and distributed in 10 nm bins. The SVs that were at the maximal distance of 10 nm were considered as docked vesicles. SVs were also distributed in 5 nm bins to distinguish fully primed and tightly docked SVs (0–5 nm) from loosely docked SVs (5–10 nm). The data were analyzed blind to the genotype and treatment, and the images were analyzed using Image J 1.43 m and Origin 8.0 software.

### Drug application in “in vivo” experiments

“In vivo” experiments were carried out with 3-month-old male *Fmr1*KO mice and littermate WT. Animal were housed in individual cages until the end of the experiment. Mice were kept on a 12-h light/dark cycle with constant ambient temperature (22 ± 0.5 °C) and humidity (55 ± 3%). They had food and water available ad libitum.

The mice were intraperitoneally (i.p.) injected with mGluR4 PAM (VU 0155041, 5 mg/Kg) [31], or the saline vehicle alone, 2 h prior to performing the behavioral experiments. Four experimental groups were established: WT and *Fmr1*KO mice injected with either saline or VU 0155041. Animals from each genotype were randomly allocated to one of two groups (saline or VU 0155041). For rotarod, elevated path, classical eyeblink conditioning and vestibular stimulation and recording of the VOR, two independent cohorts of mice were used, dividing each cohort in the four experimental groups. For skilled reaching and social interaction tests, five independent cohorts of mice were used, dividing each cohort in the four experimental groups.

### Rotarod

We used an accelerating rotarod treadmill (Ugo Basile, Varese, Italy). A mouse was placed on the rod and tested at 2–20 rpm (of increasing speed) the first day, for a maximum of 5 min. Mice were tested twice with an interval of one hour during the same first day. Animals were re-tested 24 and 48 h later. Between these trials, mice were allowed to recover in their cages. The total time that each animal remained on the rod was computed as latency to fall (s), recorded automatically by a trip switch located under the floor of each rotating drum. Results were evaluated by averaging the data collected from each of the 4 trials [34].

### Elevated path

The elevated path consisted of a 40 cm long, 5 cm wide bar located 60 cm over a soft cushion. Each mouse was placed in the center of the elevated bar and allowed a maximum of 40 s to reach one of the platforms (12 × 12 cm) located at each end of the bar. The time spent to reach one of the two platforms was quantified [34].

### Skilled reaching test

Mice were food deprived (70% of normal intake) before performing the test [35]. Briefly, a Plexiglas reaching box was used (20 cm long × 8 cm wide × 20 cm high) with a 1 cm wide vertical slit in the front of the box. Animals have to reach the palatable food pellet (20 mg dustless precision sucrose-flavored food pellets, F0071: Bio Serv) on a shelf (4 cm wide × 8 cm long) in front of the vertical slit. There is a 4 mm gap between the platform that holds the food pellets and the slit that prevents the mice from sliding the food pellets toward them. Mice were habituated to food pellets (20 min) for two days prior to testing. Those animals that did not even attempt to grasp the pellet were discarded from the study. In the first day the food pellets were put in the box, and during the second day on the shelf. The 5 days of testing consisted of a 20 min session each day. Pellet grasping and retrieval was scored as a success, whereas pellet displacement without retrieval or grasping the pellet, dropping it before it was retrieved, and pellet displacement were considered an unsuccessful attempt. Results were represented as the ratio of total successes/total attempts.

### Animal’s preparation for chronic recording experiments

Animals were deeply anesthetized with 1.2% isoflurane supplied from a calibrated Fluotec 5 (Fluotec-Ohmeda, Tewksbury, MA, USA) vaporizer at a flow rate of 0.8 L/min oxygen. The gas was delivered via an anesthesia mask adapted for mice (David Kopf Instruments, Tujunga, CA, USA). Animals were implanted with bipolar recording electrodes in the left orbicularis oculi muscle and with bipolar stimulating electrodes on the ipsilateral supraorbital nerve. Implanted electrodes were made of 50 µm, Teflon-coated, annealed stainless steel wire (A-M Systems, Carlsborg, WA, USA), with their tips bent as a hook to facilitate a stable insertion in the orbicularis oculi muscle and bared of the isolating cover for 0.5 mm. Two 0.1-mm bare silver wires were affixed to the skull as ground. The 6 wires were soldered to a six-pin socket and the socket fixed to the skull with the help of 2 small screws and dental cement. In a final surgical step, a holding system was fixed to the skull for its proper stabilization during head rotation and eye movement recordings. Further details of this chronic preparation have been detailed elsewhere [36, 37].

### Classical eyeblink conditioning

Experimental sessions started a week after surgery and were carried out with six animals at a time. Animals were placed in individual and ventilated plastic chambers (5 × 5 × 10 cm) located inside a larger Faraday box (35 × 35 × 25 cm). For classical eyeblink conditioning, we used a delay paradigm consisting of a 350 ms tone (2.4 kHz, 85 dB) as a conditioned stimulus (CS) followed at its end by an electrical shock (0.5 ms, 3 × threshold, cathodal, square pulse), applied to the supraorbital nerve, as an unconditioned stimulus (US).

A total of two habituation and 10 conditioning sessions were carried out for each animal. A conditioning session consisted of 60 CS-US presentations and lasted for about 30 min. Paired CS-US presentations were separated at random by 30 ± 5 s. For habituation sessions, the CSs were presented alone, also for 60 times per session, at intervals of 30 ± 5 s [36].

The electromyographic (EMG) activity of the orbicularis oculi muscle was recorded with Grass P511 differential amplifiers (Grass-Telefactor, West Warwick, RI, USA), at a bandwidth of 0.1 Hz-10 kHz.

### Vestibular stimulation and recording of the VOR

For vestibular stimulation, a single animal was placed on a home-made turning-table system. Its head was immobilized with the help of the implanted holding system, while the animal was allowed to walk over a running wheel. Table rotation was carried out by hand following a sinusoidal display in a computer screen. Actual rotation of the table was recorded with a potentiometer attached to its rotating axis. The animal was rotated by ± 20 deg at three selected frequencies (0.1, 0.3, and 0.6 Hz) for about ten cycles with intervals of 5 s between frequencies. The mouse right eye was illuminated with a red cold light attached to the head holding system. Eye positions during rotation were recorded with a fast infrared CCD camera (Pike F-032, Allied Technologies, Stadtroda, Germany) affixed to the turning table, at a rate of 50 or 100 pictures/s.

Recordings of head rotations and eye positions were synchronized and analyzed offline with an analog/digital converter (CED 1401 Plus, Cambridge, England) for gain and phase. Gain was computed as the averaged angular displacement of the eye (peak-to-peak) divided by the angular displacement of the head evoked experimentally. VOR gain in mice is usually <<1 [38, 39]. Phase was determined as the averaged angular difference (in degrees) between peak eye position vs. peak head position [37, 38].

### Social interaction test

To evaluate social interaction the subject (male adult mice) was allowed to move freely in a neutral cage (69 × 41 × 37 cm), containing a small inverted grid box on each side. For habituation each mouse was placed in the cage for 5 min with both boxes empty to discard mice that preferred either half of the cage. In the sociability session, a strange juvenile mouse that had not previously been encountered was put underneath one of the grid inverted boxes. In the social novelty session, another stranger mouse was put inside the previously empty box. Each session lasted 10 min, and all the experiments were video recorded and analyzed by the researcher. The unfamiliar mouse was the same for each cohort of mice (5–6 test mice for each of the four experimental conditions). The test estimates the time spent by the subject in close proximity to the juvenile mice, including the time during which the subject oriented its nose to the occupied box and sniffed in a distance < 1 cm, and the time the subject touched the occupied box. The interaction time has been used to calculate the Discrimination Index (DI). DI = (X_2_-X_1_)*100/(X_2_ + X_1_), where X_2_ and X_1_ is the interaction time with the mouse-containing and empty cages, respectively in the sociability test; whereas X_2_ and X_1_ is the interaction time with the unfamiliar and familiar mouse, respectively, in the social novelty test.

### Data collection and analysis of “in vivo” experiments

Recorded videos from each test were analyzed blind to the genotype and treatment. For classical eyeblink conditioning, unrectified EMG activity of the orbicularis oculi muscle, and 1-V rectangular pulses corresponding to CS and US presentations were stored digitally in a computer through an analog/digital converter (CED 1401-plus) for quantitative off-line analysis. Collected data were sampled at 10 kHz for EMG recordings, with an amplitude resolution of 12 bits. A computer program (Spike2 from CED) was used to display the EMG activity of the orbicularis oculi muscle. For experiments involving the VOR, head and eye positions were stored digitally in the same an analog/digital converter and processed off-line using a Matlab script (MathWorks, Natick, MA) with home-made tracking program.

### Experimental design and statistical analysis

**Fig. 1:**
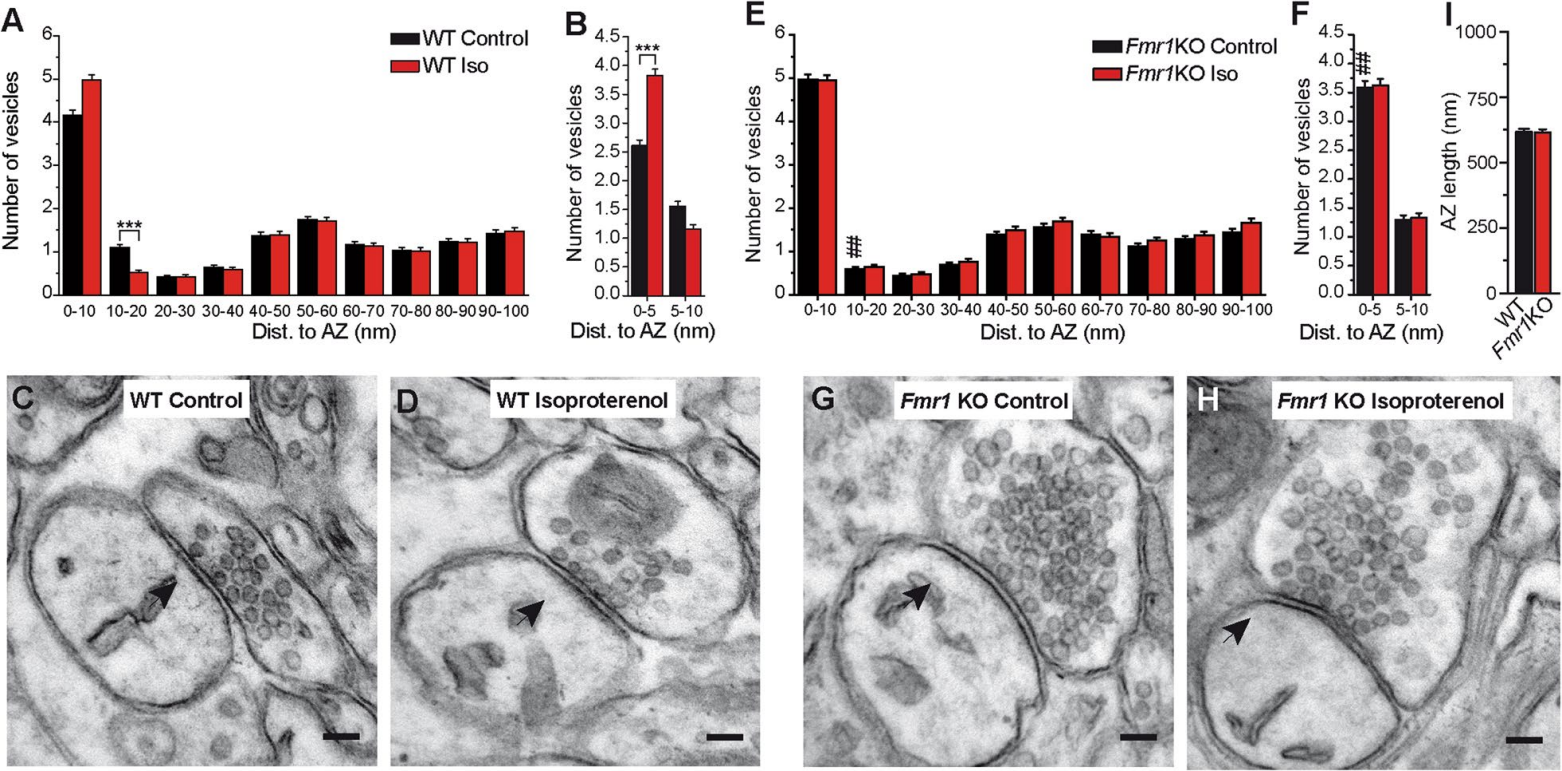
Enhanced SV docking of *Fmr1*KO synapses and lack of an effect of isoproterenol. Isoproterenol (100 µM, 10 min) increases SV docking in WT (**a, c, d**) but not in *Fmr1*KO (**e, g, h**) PF-PC synapses. Isoproterenol induces an increasing trend in the number of docked vesicles (0–10 nm) in WT (**a**) (n = 250/3 and 203/3 synapses/mice: *P* = 0.059) at the expense of SVs within 10–20 nm, which decreased in number (***P < 0.001). These changes are absent in *Fmr1*KO slices (**e**) (n = 238/4 and 224/4 synapses/mice, 0–10 nm: *P* = 0.997; 10–20 nm: *P* = 0.988). Note the increase in SV at 10–20 nm in *Fmr1*KO control as opposed to WT control slices (^##^*P* < 0.01). **c, d, g, h** Electron microscopy of PF-PC synapses. (B,F) The effect of isoproterenol on the SV distribution: WT (0–5 nm: ***P < 0.001); *Fmr1*KO (0–5 nm: *P* > 0.05). *Fmr1*KO control vs WT control at 0–5 nm (^##^*P* < 0.01). (**i**), Active Zone (AZ) length of WT (n = 250) and *Fmr1* KO (n = 238) synapses (*P* > 0.05). The values represent the mean ± S.E.M. Scale bar in (**c, d, g, h**) 100 nm. Generalized Linear Mixed Models (GLMM) with genotype and treatment as fixed effects, and animal subject as a random effect. P-values for fixed effects and their interactions were obtained with type II Wald Chi-Square tests and post-hoc tests were adjusted with the Tukey HSD method (**a, b, e, f**) and unpaired student’s *t* test in (**i**). n is the number of synapses, many synapses were analyzed per slice and several slices per mice

The appropriate sample size was previously computed depending on the power of each test using Statgrahics Centurion XVII.2. For statistical analysis GraphPad InStat v2.05a (GraphPad Software, San Diego, CA, USA); SigmaPlot 10 (Systat Software Inc., San Jose, CA USA) and OriginPro 8.0 (OriginLab Corporation, Northampton, MA, USA) were used. For comparison between two sets of data unpaired two-tailed Student t test was used or the Welch test when the variances of the populations were significantly different. When more than two sets of data were compared, one-way ANOVA (post hoc Holm-Sidak’s method), two-way ANOVA (Bonferroni’s or Tukey’s post hoc tests) or two-way repeated measures ANOVA followed by all paired multiple comparisons procedure Holm-Sidak’s or LSD post hoc tests, were used. When variances of the populations were significantly different the Kruskal–Wallis test was used and Dunn’s as post hoc test. For details see [36]. To avoid a potential problem of pseudoreplication in the analysis of SV distribution (Fig. 1), the number of SVs within a certain distance of the synapse were modelled with Generalized Linear Mixed Models (GLMM) using the Poisson distribution, with genotype and treatment as fixed effects, and animal subject as a random effect. P-values for fixed effects and their interactions were obtained with type II Wald Chi-Square tests and post-hoc tests were adjusted with the Tukey HSD method. The data are represented as raw data and mean and in some cases as the mean ± S.E.M.: *P < 0.05, **P < 0.01, ***P < 0.001. Differences were considered statistically significant when *P* < 0.05 with a confidence limit of 95%.

## Results

### *Fmr1*KO synapses have more docked SVs than WT synapses and they are insensitive to isoproterenol

We recently found an enhanced SV docking at cerebrocortical *Fmr1* KO synapses that prevents a further increase by the activation of β-ARs with isoproterenol [13]. First, we tested whether these changes also occur at PF-PC synapses. We observed an increasing trend in the number of docked vesicles at PF-PC WT synapses (those located within 10 nm of the AZ membrane) after the exposure to isoproterenol (Generalized Linear Mixed Model, GLMM, *P* = 0.048 for interaction genotype-treatment; followed by Tukey HSD multiple comparisons test, *P* = 0.059, Fig. 1a, c, d) at the expense of SVs within 10–20 nm, which decreased in number (***P < 0.001, Fig. 1a). Notably, we also observed a increasing trend in the number of docked SVs at PF-PC *Fmr1*KO synapses in the basal state compared to WT synapses (*P* = 0.072) and isoproterenol failed to significantly increase this parameter (*P* = 0.997, Fig. 1e, g, h). Moreover, isoproterenol did not change the SVs at a distance of 10–20 nm from the AZ membrane in *Fmr1*KO synapses either (*P* = 0.988, Fig. 1f).

Loosely docked and primed SVs located 8 nm from the AZ membrane due to the partial zippering of SNARE complexes were distinguished from the tightly docked and fully primed SVs in which SNARE complex zippering has progressed much further, bringing the SVs closer to the AZ membrane (0–5 nm) [40–43]. There were more SVs within 0–5 nm of the AZ membrane in WT slices following exposure to isoproterenol (GLMM, *P* = 0.004 for interaction genotype-treatment; followed by Tukey HSD multiple comparisons test (***P < 0.001). Moreover, *Fmr1*KO synapses had more SVs within 5 nm than WT synapses (^##^*P* < 0.01, Fig. 1f) and isoproterenol failed to further increase this parameter (*P* = 0.951). No change in the active zone (AZ) length was observed between WT and *Fmr1* KO synapses, [unpaired Student’s *t* test, t(913) = 0.43, P > 0.05, Fig. 1i]. Thus, *Fmr1* KO synapses have more tightly docked and fully primed SVs than WT synapses, and the ability of β-ARs to further increase this parameter is prevented.

### The lack of FMRP increases aEPSC frequency and prevents isoproterenol induced potentiation despite normal β-AR expression and cAMP generation

**Fig. 2:**
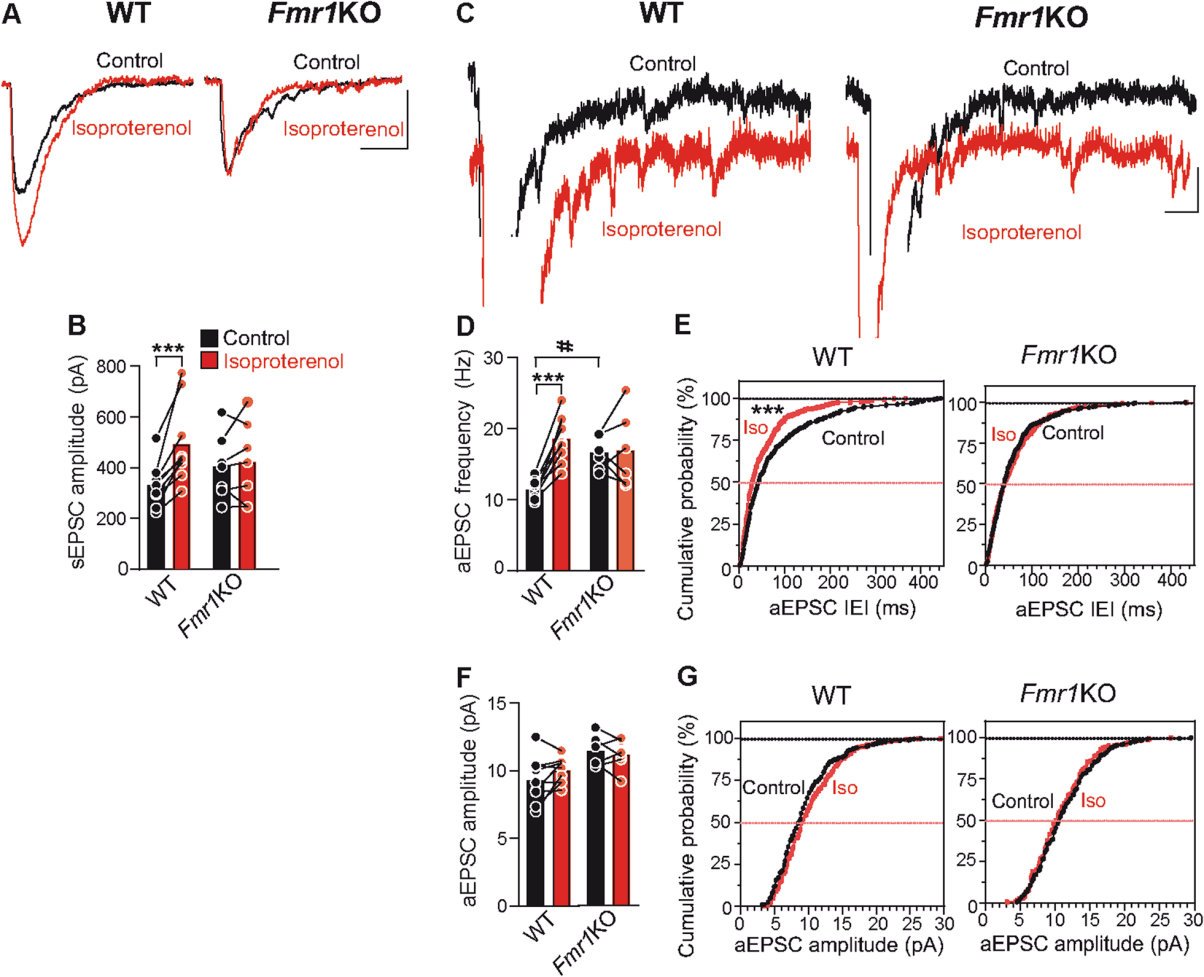
An increased aEPSC frequency and absence of isoproterenol induced potentiation at *Fmr1*KO synapses. **a** Isoproterenol (100 μM, 10 min) enhances the sEPSCs recorded in the presence of Sr^2+^ (2.5 mM) in WT but not in *Fmr1*KO slices. **b** Quantification of the effects of isoproterenol on the sEPSC amplitude in WT (n = 9, ***P < 0.001 compared to control values) and in *Fmr1*KO slices (n = 6, *P* > 0.05). **c** Individual traces showing asynchronous release events in control (black) and isoproterenol exposed (red) WT and *Fmr1*KO slices. **d, f** Quantification of the isoproterenol induced changes in aEPSC frequency (**d**) in WT (n = 306 events/9 slices and, n = 502 events/9 slices: ***P < 0.001) and *Fmr1*KO slices (n = 305 events/6 and n = 312 events/6 slices: *P* > 0.05 comparing to control values; ^#^P < 0.05 comparing to control aEPSC frequency in WT slices), as well as in amplitude (**f**) in WT (*P* > 0.05) and in *Fmr1*KO slices (*P* > 0.05). **e, g** Cumulative probability plots of isoproterenol induced changes in aEPSC frequency (inter event interval, IEI) (**e**) in WT (***P < 0.001) and *Fmr1*KO slices (*P* > 0.05), and amplitude (**g**) in WT (*P* > 0.05) and *Fmr1*KO slices (*P* > 0.05). Bar graphs show raw data and the mean. Scale bars in (**a, c**) represent 100 pA and 10 ms, and 25 pA and 10 ms, respectively. Two-way ANOVA followed by Tukey test in (**b, d, f**). Kolmogorov–Smirnov test in (**e, g**). Several slices per mice were prepared

We asked whether enhanced SV docking results in an increase in spontaneous neurotransmitter release. However, as it is not possible to distinguish PF from climbing fiber miniature excitatory postsynaptic currents (mEPSCs) [44] we measured asynchronous release evoked by PF stimulation in the presence of Sr^2+^. Under these conditions, asynchronous release represents single release events from PF [44]. In WT slices, isoproterenol increased synchronous release (two-way ANOVA followed by Tukey’s multiple comparison test, F(3, 26) = 18.92, ***P < 0.001, Fig. 2a, b) and the frequency (two-way ANOVA followed by Tukey’s multiple comparison test, F(3, 26) = 8.49, ***P < 0.001, Fig. 2c–e) but not the amplitude (two-way ANOVA followed by Tukey’s multiple comparison test, F(3, 26) = 0.30, *P* > 0.05, Fig. 2c, f, g) of asynchronous release. However, isoproterenol failed to enhance sEPSCs at *Fmr1KO* slices (*P* > 0.05, Fig. 2a, b), and as the frequency of aEPSCs in *Fmr1KO* mice was higher than in WT slices (^#^*P* < 0.05, Fig. 2d), this further prevented the potentiation by isoproterenol (*P* > 0.05, Fig. 2d, e).

We assessed whether the failure of isoproterenol to potentiate SV docking and neurotransmitter release at *Fmr1*KO synapses was a consequence of changes in β-AR expression and/or activity, which was examined in a preparation of cerebellar nerve terminals (synaptosomes). The spontaneous release of glutamate from WT cerebellar synaptosomes increased in the presence of isoproterenol (two-way ANOVA followed by Tukey’s multiple comparison test, F(3, 24) = 14.33, ***P < 0.001, Additional file 1: Figure S1*A,C*). However, *Fmr1*KO synaptosomes showed an increase in spontaneous release under basal conditions (^#^*P* < 0.05, Additional file 1: Figure S1*C*) but this was not further potentiated by isoproterenol (*P* > 0.05, Additional file 1: Figure S1*B,C*). Thus, *Fmr1*KO cerebellar synaptosomes recapitulate the occlusion phenotype evident in *Fmr1*KO slices.

The absence of β-AR mediated potentiation of spontaneous release in *Fmr1*KO synaptosomes could be due to weaker expression of this receptor. However, β1-AR expression was similar in *Fmr1*KO and WT synaptosomes when assessed in western blots (unpaired Student’s t test, t(4) = 0.61, *P* > 0.05, Additional file 1: Figure S1D,E). We also assessed β-AR expressing synaptosomes by immunofluorescence using antibodies against β_1_-AR and synaptophysin as a marker of SVs. There were a similar number of *Fmr1*KO and WT cerebellar synaptosomes labeled for synaptophysin that also expressed β_1_-AR synaptosomes (unpaired Student’s t test, t(136) = 1.29, *P* > 0.05, Additional file 1: Figure S1*F,G,H*). These data indicate that a change in β_1_-AR expression is not responsible for the failure of isoproterenol to potentiate spontaneous release in *Fmr1* KO synaptosomes. β-ARs activate Adenylyl Cyclase (AC) and generate cAMP, which activates downstream signals to potentiate spontaneous release [45]. The lack of isoproterenol induced potentiation was not due to loss of receptor function as there was a similar β-AR mediated increase in cAMP in *Fmr1*KO synaptosomes as that in WT synaptosomes (unpaired Student’s t test, t(27) = 0.193, *P* > 0.05, Additional file 1: Figure S1*I*), reinforcing the idea that potentiation mediated by β-ARs is prevented at *Fmr1* KO synapses.

### Parallel fiber LTP is absent in PF-PC *Fmr1*KO synapses

**Fig. 3:**
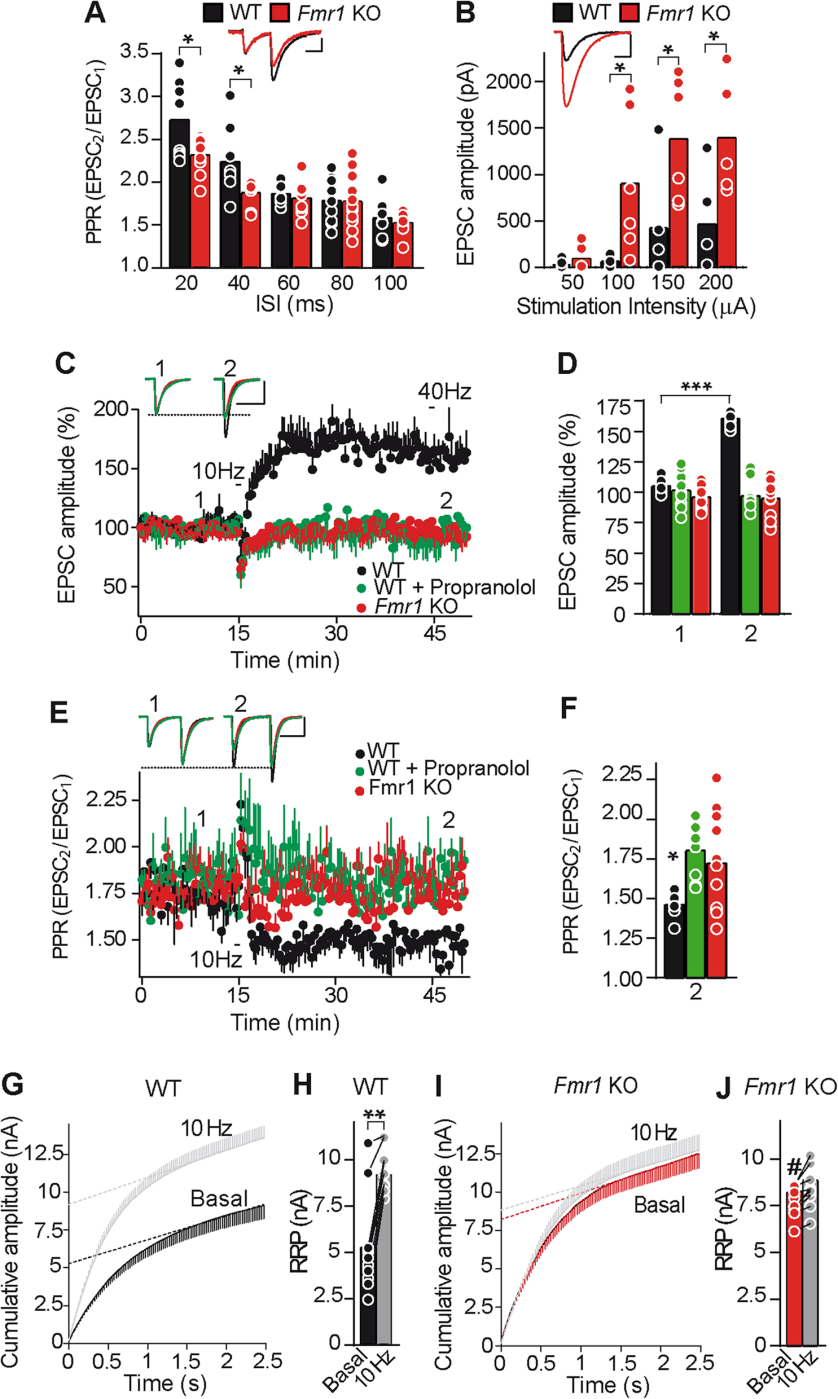
*Fmr1* KO synapses have increased RRP size and lack cerebellar PF-PC LTP. **a** Changes in the PPR (mean of 6 consecutive pairs of stimuli delivered at 0.05 Hz) compared to WT at different inter-interval stimuli (ISI) (WT: n = 8 cells, 8 slices, 3 mice. *Fmr1* KO: n = 9 cells, 9 slices, 3 mice. Scale bars: 100 pA, 20 ms), **b** changes in synaptic efficacy (mean of 6 consecutive EPSCs delivered at 0.05 Hz) compared to WT at different stimuli intensity (WT: n = 6 cells, 6 slices, 3 mice. *Fmr1*KO: n = 7 cells, 7 slices, 3 mice). Scale bars: 500 pA, 1 ms. **c** Changes in EPSC amplitude induced by a 10 Hz stimulation. **d** Quantification of EPSCs (mean of 6 consecutive EPSCs delivered at 0.05 Hz) 30 min after stimulation (2) compared to the respective values before stimulation (1): WT, (n = 9 cells/9 slices/5 mice); WT/propranolol (100 μM, 30 min, n = 10 cells/10 slices/6 mice); *Fmr1* KO (n = 12 cells/12 slices/9 mice). Scale bars: 50 pA, 10 ms. **e** Changes in the PPR. **f** Quantification of the changes in PPR (mean of 6 consecutive pairs of stimuli delivered at 0.05 Hz) compared to the respective values before stimulation: WT (n = 9 cells/9 slices/5 mice); WT/propranolol (100 μM, 30 min, n = 10 cells/10 slices/6 mice); *Fmr1* KO (n = 12 cells/12 slices/9 mice). Scale bars: 20pA, 10 ms. **g, i** Cumulative EPSC amplitudes in WT and *Fmr1*KO slices before (basal) and 30 min after LTP induction by 10 Hz stimulation (10 Hz). RRP size was calculated from the cumulative amplitude plots as the y-intercept from a linear fit of the steady-state level attained during a train of 100 stimuli at 40 Hz (as see in **c**). **h, j** Quantification of the RRP size: WT (n = 11 cells/11 slices/5 mice: **P < 0.01); *Fmr1*KO (n = 13 cells/13 slices/6 mice: *P* > 0.05, comparing 10 Hz vs basal values). RRP size WT basal vs *Fmr1*KO basal (^#^*P* < 0.05). Bar graphs show raw data and the mean. *P < 0.05, **P < 0.01, ***P < 0.001, ^#^*P* < 0.05, unpaired Student’s t test or Welch test when the variances of the populations were significantly different

An increase in the number of releasable vesicles at PF-PC *Fmr1*KO synapses should decrease the paired-pulse ratio (PPR). PPR was reduced at the interstimulus interval (ISI) of 20 ms (unpaired Welch test, t(9) = 2.35, *P < 0.05) and 40 ms (unpaired Welch test, t(8) = 2.49, *P < 0.05). Although no change was observed at higher ISIs (Fig. 3A). An increase in the number of releasable vesicles at PF-PC *Fmr1* KO synapses should also increase the synaptic efficacy (averaged EPSC amplitude including failures) measured at different stimulation intensities. *Fmr1*KO synaptic efficacy was not altered at 50 μA (unpaired Welch test, t(5) = 1.09, *P* > 0.05) but increased at 100 μA (unpaired Welch test, t(5) = 2.64, *P < 0.05), 150 μA (unpaired Student’s t test, t(10) = 2.59, *P < 0.05) and 200 μA (unpaired Student’s t test, t(10) = 2.75, *P < 0.05, Fig. 3B) compared to WT.

Presynaptic LTP at PF-PC synapses depends on a Ca^2+^ mediated increase in presynaptic cAMP [30, 46] and on the RIM1α protein, a Rab-3 interacting molecule [47]. We recently found that PF-PC LTP requires a β-adrenergic receptor (β-AR) dependent increase in SV docking and an increase in the RRP size [48], events that contribute to enhanced neurotransmitter release [45, 48]. As presynaptic responses of β-ARs are absent at *Fmr1*KO PF-PC synapses, we assessed whether PF-PC LTP was lost at these synapses. PF-PC LTP can be induced by stimulating PFs at 10 Hz for 10 s [47] and it is expressed as a long lasting increase in EPSC amplitude (unpaired Student’s t test, t(16) = 5.04, ***P < 0.001, Fig. 3C,D). The presynaptic origin of this LTP was evident by the decreased paired-pulse ratio (PPR; unpaired Welch test, t(11) = 2.46, *P < 0.05, Fig. 3E,F), and the β-AR receptor antagonist propranolol prevented PF-PC LTP in WT slices (unpaired Welch test, t(14) = 0.38, *P* > 0.05, Fig. 3C,D). However, stimulation of PFs at 10 Hz for 10 s failed to induce LTP at PF-PC *Fmr1*KO synapses (unpaired Welch test, t(17) = 0.13, *P* > 0.05, compared to the baseline, Fig. 3C,D).

PF-PC LTP involves an increase in the RRP size [48] and hence, we determined if a change in the RRP size could explain the lack of LTP at *Fmr1*KO PF-PC synapses. An increase in the RRP size was evident 30 min after LTP induction at WT PF-PC synapses (unpaired Student’s t test, t(23) = 3.59, **P < 0.01, Fig. 3G,H). However, the basal RRP was larger in *Fmr1* KO synapses than in WT slices (unpaired Student’s t test, t(22) = 2.54, *P < 0.05) and it was not further enhanced by stimulation at 10 Hz (unpaired Student’s t test, t(25) = 0.49, *P* > 0.05, Fig. 3G,H,I,J). As such, *Fmr1*KO synapses have a larger RRP under basal conditions that impedes LTP.

### Decreasing extracellular Ca^2+^ reduces asynchronous release and rescues parallel fiber LTP in *Fmr1*KO slices

**Fig. 4:**
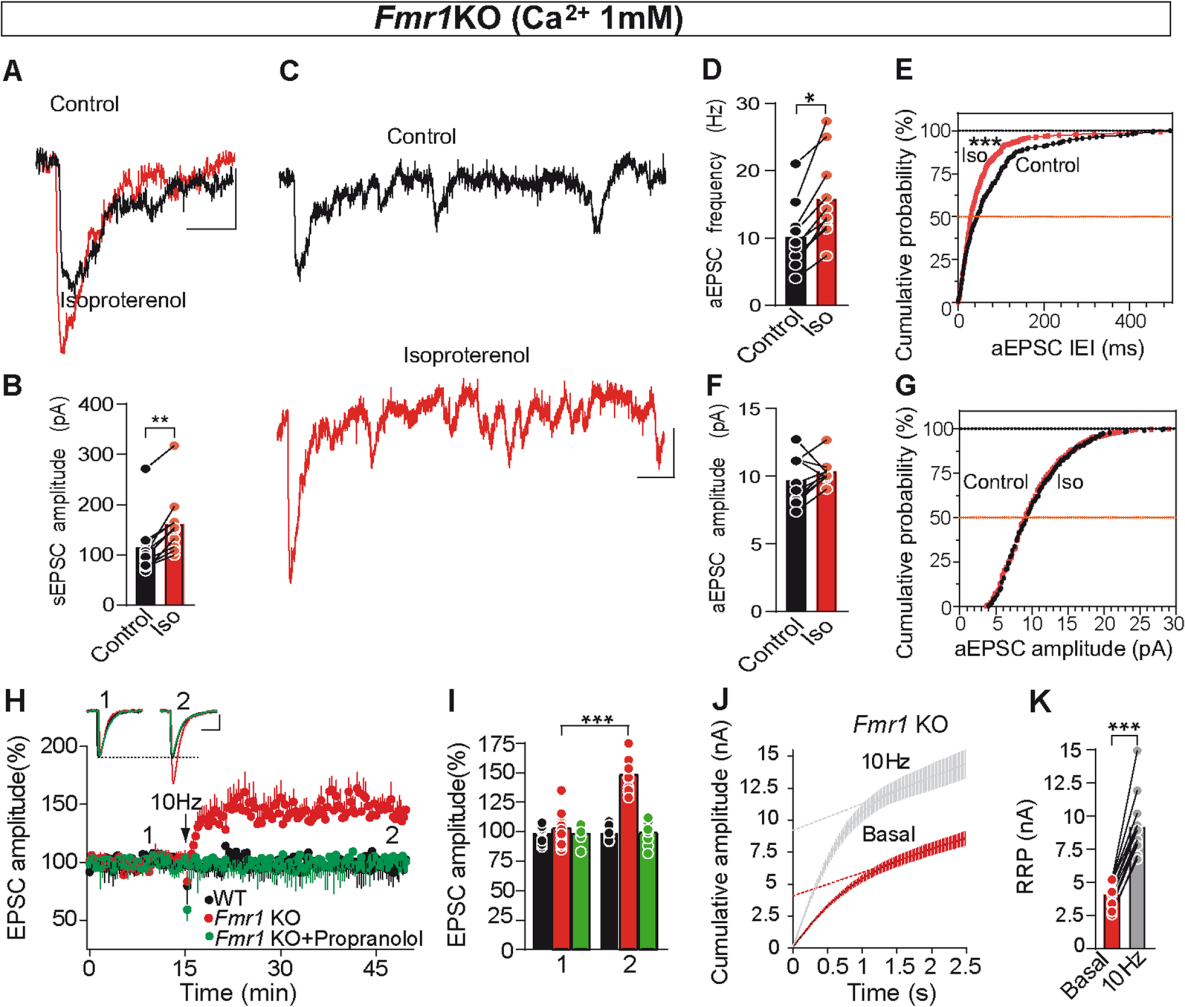
Reducing extracellular Ca^2+^ reduces asynchronous release and rescues isoproterenol-induced potentiation and PF-PC LTP at *Fmr1*KO slices. **a** Isoproterenol (100 μM, 10 min) enhances the sEPSC amplitude recorded in the presence of Sr^2+^ (1.0 mM). **b** Quantification of the effects of isoproterenol on sEPSC amplitude: (n = 6: **P < 0.01, unpaired Student’s t test). **c** Individual traces showing asynchronous release events in control (black) and after exposure to isoproterenol (red). **d, f** Quantification of the isoproterenol induced changes in aEPSC frequency (**d**) (n = 305 events/10 slices and n = 474 events/10 slices: two-way ANOVA followed by Tukey, *P < 0.05) and in aEPSC amplitude (**f**) (*P* > 0.05, unpaired Student’s t test). **e, g** Cumulative probability plots of isoproterenol induced changes in aEPSC frequency (inter event interval, IEI) (**e**) (***P < 0.001) and aEPSC amplitude (**g**) (*P* > 0.05, Kolmogorov–Smirnov test). **h** PF LTP was re-established in *Fmr1*KO slices, yet no LTP was induced in WT slices in the presence of 1 mM Ca^2+^. Experiments in *Fmr1*KO slices were also performed in the presence of the β-AR antagonist propranolol (100 µM, added 30 min prior to LTP induction). **i** Changes in EPSC amplitude (mean of 6 consecutive EPSCs delivered at 0.05 Hz) 30 min after stimulation (2) compared to basal (1): in *Fmr1*KO (n = 14 cells/14 slices/7 mice: ***P < 0.001, unpaired Student’s *t* test); in *Fmr1*KO slices treated with propranolol (n = 9 cells/9 slices/4 mice: *P* > 0.05, Welch test); and in WT (n = 10 cells/10 slices/4 mice: *P* > 0.05, unpaired Student’s *t* test). **j** Cumulative EPSC amplitudes (in the presence of 1 mM Ca^2+^) before and 30 min after LTP induction in *Fmr1*KO slices. RRP size was calculated from the cumulative amplitude plots as the y-intercept from a linear fit of the steady-state level attained during a train of 100 stimuli at 40 Hz. **k** Quantification of the RRP in *Fmr1*KO slices (n = 12 cells/12 slices/4 mice and n = 12 cells/12 slices/5 mice, before and after LTP induction: ***P < 0.001, Welch test). Bar graphs show raw data and the mean. Scale bar in **a, c** 10 pA and 100 ms; and in **h** 50 pA and 30 ms

One presynaptic change at *Fmr*1KO synapses is the loss of functional Ca^2+^-activated K^+^ channels, with the result of action potential, AP, broadening and enhanced presynaptic Ca^2+^ influx [12]. Thus, we tested whether reducing [Ca^2+^]_e_ from 2.5 to 1 mM re-established isoproterenol mediated potentiation in *Fmr1*KO synapses. When slices were maintained at 1 mM [Ca^2+^]_e_, isoproterenol increased the sEPSCs amplitude (unpaired Student’s t test, t(18) = 3.56, **P < 0.01, Fig. 4A,B). Moreover, the aEPSC frequency of *Fmr*1KO synapses decreased to values similar to those of WT synapses in 2.5 mM [Ca^2+^]_e_ (two-way ANOVA followed by Tukey’s multiple comparison test, F(3, 34) = 7.17, *P* > 0.05, Fig. 4D), and consequently, exposure to isoproterenol increased the aEPSC frequency (*P < 0.05, Fig. 4C,D,E) with no change in aEPSC amplitude (unpaired Student’s t test, t(18) = 0.02, *P* > 0.05, Fig. 4C,F,G). When the [Ca^2+^]_e_ was lowered to 1 mM, PF-PC LTP was also re-established at *Fmr1*KO slices (unpaired Student’s t test, t(26) = 4.49, ***P < 0.001, Fig. 4H,I*)*, even though LTP was not supported in WT slices at this [Ca^2+^]_e_ (unpaired Student’s t test, t(18) = 0.05, *P* > 0.05, Fig. 4H,I), consistent with earlier reports on the sensitivity of PF-PC LTP to decreases in [Ca^2+^]_e_ [32]. Interestingly, the rescued LTP in *Fmr1*KO slices also exhibited other features of PF-PC LTP seen in WT synapses [48], such as its sensitivity to the β-AR antagonist propranolol (unpaired Welch test, t(10) = 0.09, *P* > 0.05, Fig. 4H,I) and the increase in RRP size (unpaired Welch test, t(15) = 4.96, ***P < 0.001, Fig. 4J,K). Indeed, reduced [Ca^2+^]_e_ counteracted the changes that led to increased SV docking and the enhanced RRP in *Fmr1*KO PF-PC synapses, decreasing asynchronous release, rescuing the presynaptic potentiation by β-ARs and PF-PC LTP.

### The mGluR4 PAM VU0155041 rescues parallel fiber LTP at *Fmr1* KO slices

**Fig. 5:**
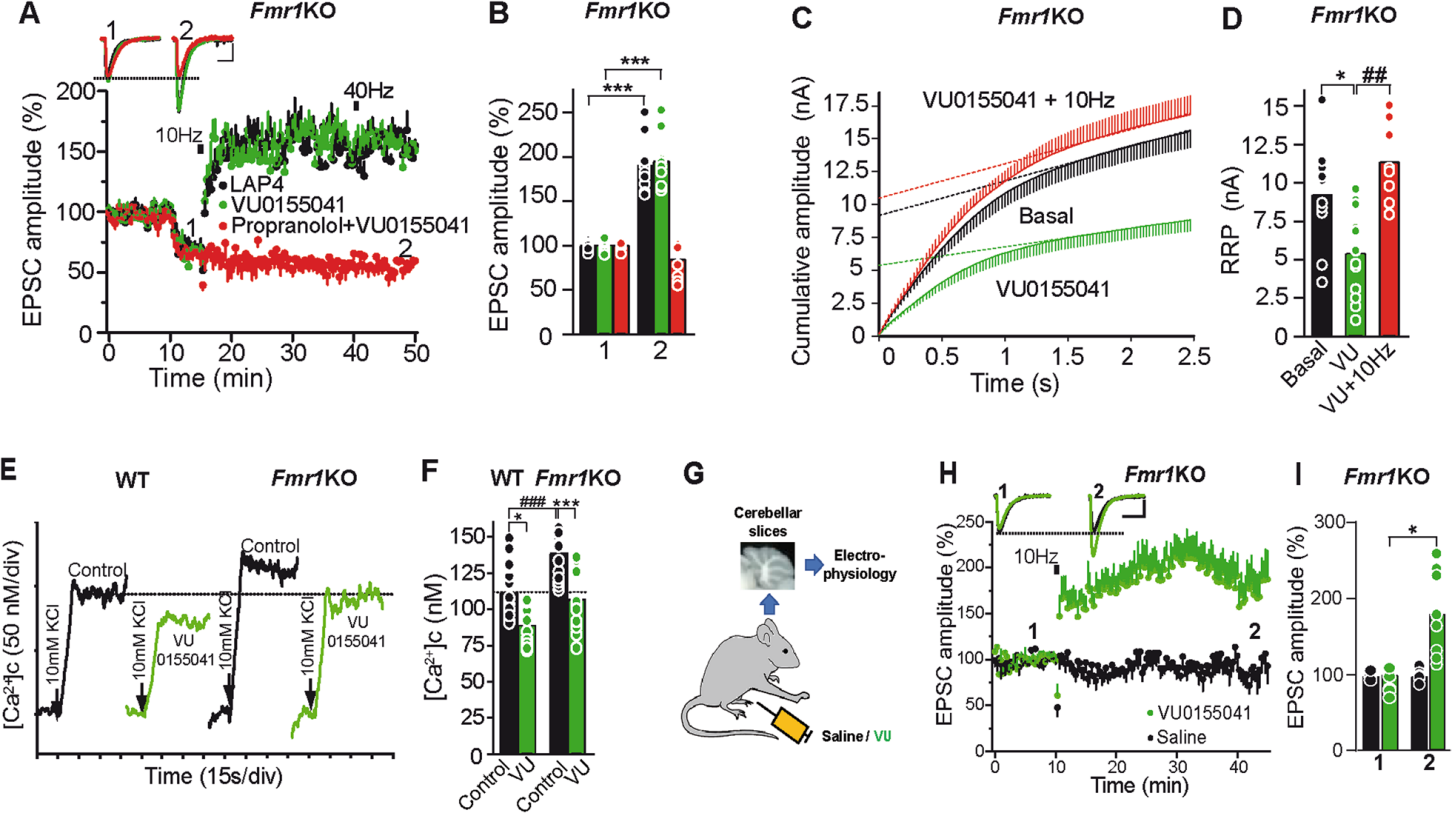
Activation of mGluR4 rescues PF-PC LTP in *Fmr1*KO slices. **a** LAP4 (40 μM) and the mGluR4 PAM VU 0155041 (100 μM) rescues PF-PC LTP in *Fmr1*KO slices. The β-AR antagonist propranolol (100 µM) was added 30 min prior to LTP induction. **b** Quantification of EPSC amplitude (mean of 6 consecutive EPSCs delivered at 0.05 Hz) 30 min after stimulation (2) compared to the respective values before stimulation (1): LAP4 (n = 10 cells/10 slices/5 mice); VU (n = 11 cells/11 slices/6 mice); propranolol + VU (n = 10 cells, 10 slices/6 mice). **c** Cumulative EPSC amplitudes in *Fmr1*KO slices in the presence or absence (basal) of VU (100 μM, 10 min) and before (VU) and 30 min after 10 Hz stimulation (VU + 10 Hz). **d** Quantification of the RRP size in the above conditions: Basal (n = 10 cells/10 slices/7 mice); VU (n = 12 cells/10 slices/7 mice: **P < 0.01 compared to basal); VU plus 10 Hz (n = 8 cells/8 slices/4 mice: ^##^*P* < 0.01, compared to VU alone). **e** VU restores Ca^2+^ dynamics to *Fmr1*KO cerebellar synaptosomes. Changes in the cytoplasmic free Ca^2+^ concentration ([Ca^2+^]_c_) in the presence and absence of VU (100 µM) added at least 5 min prior to KCl. **f** Quantification of the changes in [Ca^2+^]_c_: KCl/WT (n = 18/5 preparations); KCl/*Fmr1* KO (n = 16/5 preparations: ^###^*P* < 0.001 compared to KCl/WT); VU/KCl/WT (n = 11/4 preparations); and VU/KCl/*Fmr1*KO (n = 12/4 preparations). **g** Scheme showing the preparation of cerebellar slices from VU or saline injected *Fmr1*KO mice. **h** Response to a 10 Hz stimulation in slices from VU and saline injected *Fmr1*KO mice **i** amplitude (mean of 6 consecutive EPSCs delivered at 0.05 Hz) 30 min after stimulation (2) compared to the respective values before stimulation (1): VU (5 mg/Kg) injected *Fmr1*KO mice (n = 14 cells/14 slices/10 mice); saline injected *Fmr1*KO mice (n = 10 cells/10 slices/8 mice). Bar graphs show raw data and the mean. Scale bars in (**a, h**) 100 pA and 10 ms. Unpaired Student’s t test in (**b, d**). Two-way ANOVA followed by Tukey in (**f**). Welch test in (**i**). *P < 0.05, **P < 0.01, ***P < 0.001

Since decreasing [Ca^2+^]_e_ re-establishes LTP at *Fmr1*KO synapses, it may be possible to rescue LTP using pharmacological tools that reduce Ca^2+^ influx at PF synaptic boutons, for example through the activation of G protein coupled receptors (GPCRs). Significantly, mGluR4 reduces Ca^2+^ influx and synaptic transmission at PF synaptic boutons [49, 50], where mGluR4 is the only group III mGluR present [51]. When mGluR4s were activated by the group III mGluR agonist, L-2-amino-4-phosphonobutyric acid, L-AP4, (40 μM) there was a reduction in the EPSC amplitude and once a new baseline was established, 10 Hz stimulation induced a strong and sustained increase in the EPSC amplitude (unpaired Student’s t test, t(18) = 11.73, ***P < 0.001, Fig. 5A,B). We also tested whether VU 0155041, a potent and selective PAM of mGluR4s that is active in vivo [21, 22] rescued PF-PC LTP. VU 0155041 reduced the EPSC amplitude and after 5 min, a 10 Hz stimulation provoked a sustained increase in EPSC amplitude (unpaired Student’s t test, t(20) = 18.16, ***P < 0.001, Fig. 5A,B). The rescued LTP at *Fmr1*KO synapses required β-AR activation, as does WT LTP, and indeed, the β-AR antagonist propranolol prevented this rescue by VU 0155041 (unpaired Student’s t test, t(18) = 1.61, *P* > 0.05, Fig. 5A,B). That a mGluR4 PAM rescues PF-PC LTP does not necessarily mean that the function of this receptor is altered in *Fmr1* KO synapses. As such, we have found that the reduction of the EPSC amplitude caused by VU0155041 (100 μM) (35.6 ± 3.7%, ***P < 0.001, Welch test, t = 5.36, d.f. = 12) is similar to that in *Fmr*1 KO mice (32.5 ± 2.7%, ***P < 0.00, t = 5.01, d.f. = 20, unpaired Student’s t test) (Additional file 2: Figure S2) suggesting no changes in the expression of mGluR4 in the *Fmr1* KO. Then, the rescue by VU0155041 of PF-PC LTP could result from normalization of other parameters essential for PF-PC synaptic potentiation. Thus, VU 0155041 reduced the RRP size in *Fmr1*KO synapses under basal conditions (unpaired Student’s t test, t(20) = 2.84, *P < 0.05, Fig. 5C,D) and permitted a further increase by 10 Hz stimulation (unpaired Student’s t test, t(18) = 3.76, **P < 0.01, compared with VU 0155041 in basal conditions). Thus, the mGluR4 PAM VU 0155041 rescued normal RRP size and PF LTP in *Fmr1*KO slices.

FMRP interacts with the Ca^2+^ activated K^+^ channels that control the duration of action potentials (APs) and thus, the loss of FMRP leads to AP broadening and an ensuing increase in Ca^2+^ influx [12]. As Ca^2+^ homeostasis is altered in *Fmr1*KO mice [52] we tested whether VU 0155041 might rescue Ca^2+^ dynamics in *Fmr1*KO mice by measuring the depolarization induced change in the cytosolic Ca^2+^ concentrations ([Ca^2+^]_c_) of fura-2 loaded cerebellar synaptosomes. Synaptosomes were depolarized with a low KCl concentrations (10 mM KCl) to induce synaptic events involving Na^+^, K^+^ and Ca^2+^ channel firing which are compatible with the generation of action potentials [29]. The KCl-induced increase in [Ca^2+^]_c_ was larger in *Fmr1*KO than in WT synaptosomes (two-way ANOVA followed by Tukey’s multiple comparisons test, F(3, 56) = 17.04, ^*###*^*P* < 0.001, Fig. 5E,F), compatible with the prolonged action potentials at *Fmr1*KO synapses [12]. VU 0155041 reduced the KCl-induced change in [Ca^2+^]_c_ in WT synaptosomes (*P < 0.05, Fig. 5E,F) and it restored the KCl-induced increase in [Ca^2+^]_c_ in *Fmr1*KO to the levels of WT synaptosomes (*P* > 0.05 Fig. 5E,F). Together, these data indicate that Ca^2+^ homeostasis is deregulated in *Fmr1*KO cerebellar synaptosomes but can be restored with the mGluR4 PAM VU 0155041.

We also tested whether VU 0155041 injected “in vivo” rescue PF-PC LTP in cerebellar slices. *Fmr1*KO mice were injected (i.p.) with VU 0155041 (or the saline vehicle alone) and cerebellar slices were prepared 2 h later. PF-PC LTP was rescued in slices from VU 0155041 injected *Fmr1*KO mice (unpaired Welch’s test, t(13) = 2.932, *P < 0.05, compared to the baseline Fig. 5G,H,I) but not in those from *Fmr1*KO mice injected with saline alone (unpaired Welch’s test, t(10) = 0.37, *P* > 0.05, Fig. 5G,H,I). Similarly, VU 0155041 injected “in vivo” in adult mice (≥ 3 months) also rescued PF-PC LTP in cerebellar slices (unpaired Welch’s test, t(6) = 4.80, **P = 0.003, compared to baseline, Additional file 3: Figure S3*A,B*).

### VU0155041 ameliorates the motor learning and social deficits of *Fmr1*KO mice

**Fig. 6:**
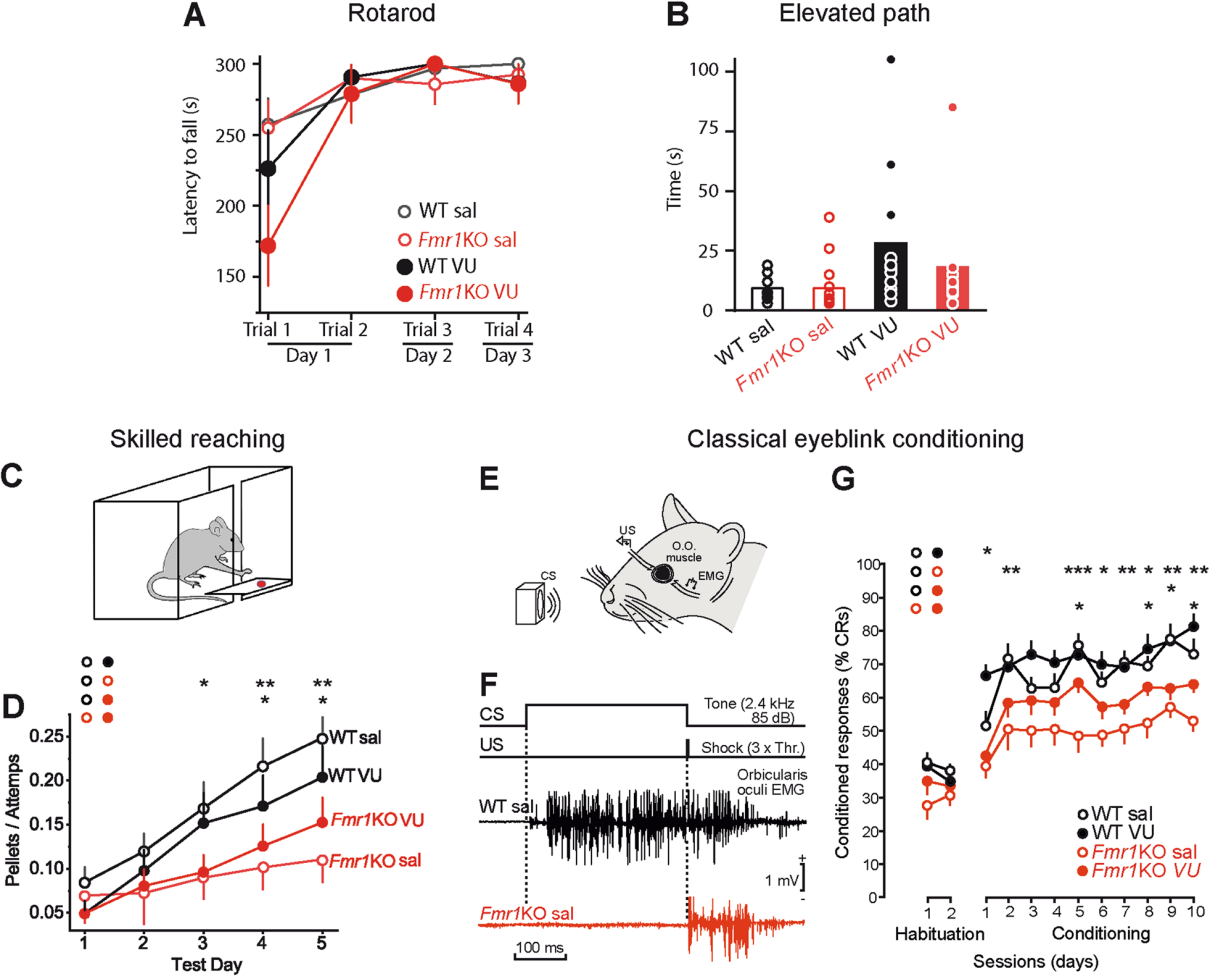
VU 155041 ameliorates skilled reaching and classical eyeblink conditioning deficits of *Fmr1*KO. **a** Latency to fall of the mouse in an accelerating rotarod. Trials 1 and 2 were performed in day 1, and 3 and 4 in days 2 and 3, respectively. WT sal (n = 11), *Fmr1*KO sal (n = 14), WT VU (n = 10) and *Fmr1*KO VU (n = 11). All comparisons to WT sal were not significant (Kruskal–Wallis followed by Dunn’s test; trial 1: H(3) = 7.538, *P* < 0.05; trial 2: H(3) = 0.915, *P* > 0.05; trial 3: H(3) = 1.754, *P* < 0.05: trial 4: H(3) = 1.101, *P* < 0.05). *Fmr1*KO VU compared to *Fmr1*KO sal was not significant at in any trials (Kruskal–Wallis followed by Dunn’s test, *P* < 0.05). **b** In the elevated path the time spent to walk from the center of a 5 cm wide bar to one of its ends is measured. WT sal (n = 11), *Fmr1*KO sal (n = 14), WT VU (n = 10) and *Fmr1*KO VU (n = 11). All comparisons to WT sal were not significant (Kruskal–Wallis followed by Dunn’s test, H(3) = 6.125, *P* > 0.05). *Fmr1*KO VU compared to *Fmr1*KO sal was not significant (Kruskal–Wallis followed by Dunn’s test, *P* < 0.05). **c** Skilled reaching test. Mice use their forelimbs to grasp and retrieve food pellets through a narrow slit. **d** Efficiency in test performance (number of pellets retrieved per attempt) in the four experimental groups: WT sal (n = 31); *Fmr1*KO sal (n = 33); WT VU (n = 31); and *Fmr1*KO VU (n = 32) during 5 days. **e** Classical eyeblink conditioning was evoked with a conditioning stimulus (CS) consisting of a 350 ms tone (2.4 kHz, 85 dB) supplied by a loudspeaker located 50 cm in front of the animal’s head. The unconditioned stimulus (US) was presented at the end of the CS, and consisted of an electrical shock (a square, cathodal pulse, lasting for 0.5 ms) presented to the left supraorbital nerve. Conditioned responses (CRs) were determined from the EMG activity of the orbicularis oculi (O.O.) muscle ipsilateral to US presentations. **f** Examples of EMG recordings collected from representative WT sal and *Fmr*1KO sal mice during the 8th conditioning session. Note the presence of a noticeable CR in the WT sal mouse and its absence in the *Fmr1*KO sal animal. **g** CRs after 10 conditioning sessions of WT sal (n = 10), *Fmr1*KO sal (n = 10), WT VU (n = 10) and *Fmr1*KO VU (n = 10). *P < 0.05, **P < 0.01, ***P < 0.001. All post-hoc comparisons were to WT sal. *Fmr1*KO VU and *Fmr1*KO sal were also compared. Two-way repeated measures ANOVA followed LSD (**d**) or by Holm-Sidak’s (**g**). The data represent the mean ± S.E.M (**a, d, g**) and raw data and the mean (**b**). n is the number of mice used

We evaluated motor coordination and learning in the rotarod. *Fmr1*KO mice showed no defects in this test that measures the time that each animal remained on the rod of and an accelerating rotarod treadmill (time to fall) (Fig. 6A), or in the elevated path that measures the time spent by a mouse placed in the center on an elevated bar to reach one of the two platforms (Fig. 6B). In both tests, all comparisons to WT sal were not significant, (Kruskal–Wallis followed by Dunn’s multiple comparison test, *P* > 0.05). *Fmr1*KO VU compared to *Fmr1*KO sal was also not significant (Kruskal–Wallis followed by Dunn’s multiple comparison test, *P* > 0.05). In order to assess the behavioral consequences of changes in basal synaptic transmission and the loss of PF-PC LTP we tested the performance of *Fmr1*KO mice in tests that evaluate motor learning. *Fmr1* KO mice display impaired motor learning in a forelimb-reaching task [20]. In this test, mice are trained to use their forelimbs to grasp and retrieve food pellets through a narrow slit (Fig. 6C), and the cerebellum contributes substantially to the coordination of the skilled movements that require speed, smoothness and precision, such as reaching to grasp movements [53, 54]. We tested whether rescuing the PF-PC LTP with VU 0155041 improved skilled reaching. After two days of habituation, the animal’s efficiency (number of pellets retrieved/number of attempts) was measured over 5 days and compared to WT sal on the same day. A deficit in skilled reaching was observed in *Fmr1*KO that receive saline injection (two-way repeated measures ANOVA followed LSD’s multiple comparisons test, F(3, 123) = 2.67; day 3: *P < 0.05, day4: **P < 0.01, day 5:**P < 0.01, compared to WT sal Fig. 6D). Interestingly, VU 0155041 administration slightly improved this task in *Fmr1*KO mice (*Fmr1*KO VU) (day 3: *P* > 0.05, day 4: *P < 0.05, day 5: *P < 0.05, compared to WT sal, Fig. 6D), while it did not alter the performance of WT mice (WT VU) (day 3: *P* > 0.05, day 4: *P* > 0.05, day 5: *P* > 0.05, compared to WT sal, Fig. 6D).

We also tested classical eyeblink conditioning and the VOR, two paradigms that specifically evaluate cerebellar-dependent motor learning related to plasticity at PF-PC synapses [17, 19, 55]. *Fmr1*KO mice show deficits in classical eyeblink conditioning [56]. In the classical eyeblink conditioning the mouse learn to associate a conditioned stimulus (CS), such as a tone, with an unconditioned stimulus (US) such as a mild electric shock to the supraorbital nerve, which evokes eyeblinks (Fig. 6E,F). As a result of the CS-US association during training the eyeblink conditioned response (CR) is progressively enhanced [57]. After 10 conditioning sessions there was a significant deficit in the number of CRs of *Fmr1*KO sal mice compared to WT sal mice (two-way repeated measures ANOVA followed by Holm-Sidak’s multiple comparison test, F(33, 396) = 1.68, **P < 0.01, Fig. 6G). VU 0155041 administration improves the performance of the *Fmr1*KO (*P* > 0.05 compared to WT sal, and P < 0.05 compared to *Fmr1*KO sal, Fig. 6G), but not that of the WT (*P* > 0.05 compared WT sal, Fig. 6G).

**Fig. 7:**
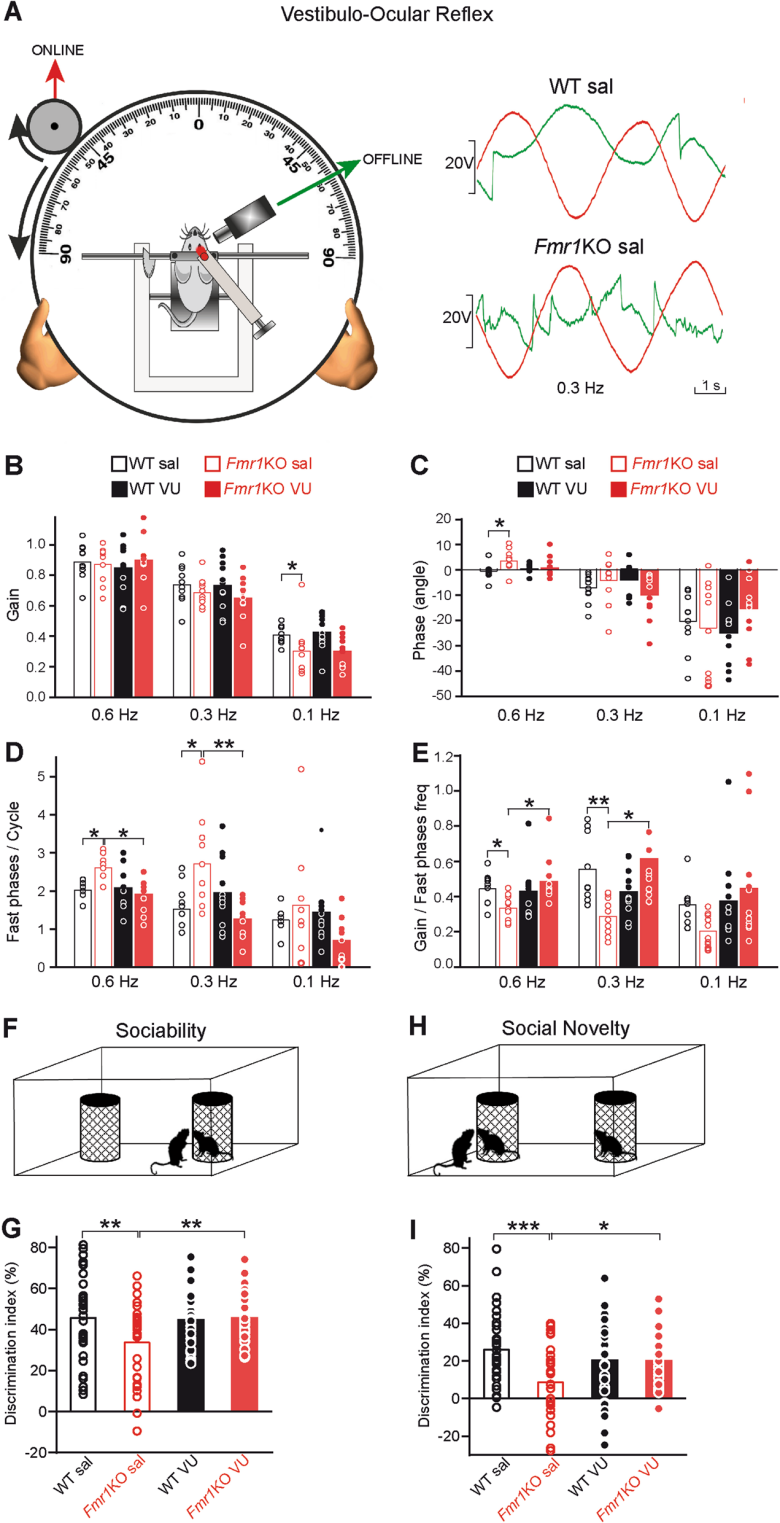
VU 0155041 ameliorates VOR and social interaction deficits of *Fmr1*KO mice. **a** Experimental design. Table rotations (red) and eye positions (green). Examples of eye movements. **b** VOR gain. 0.1 Hz: WT sal (n = 10), *Fmr1*KO sal (n = 10), *P < 0.05, WT VU (n = 11), *P* > 0.05 and *Fmr1*KO VU (n = 11), *P* > 0.05, compared to WT sal. *Fmr1*KO VU vs *Fmr1*KO sal, *P* > 0.05 (Kruskal–Wallis followed by Dunn’s test). **c** VOR phase. 0.6 Hz: *Fmr1*KO sal, *P < 0.05, WT VU, *P* > 0.05, *Fmr1*KO VU, *P* > 0.05, compared to WT sal. *Fmr1*KO VU versus *Fmr1*KO sal, *P* > 0.05 (one-way ANOVA followed by Holm-Sidak’s test). **d** Fast phases per vestibuloocular cycle. 0.6 Hz: *Fmr1*KO sal, *P < 0.05, WT VU, *P* > 0.05 and *Fmr1*KO VU, *P* > 0.05, compared to WT sal. *Fmr1*KO VU vs *Fmr1*KO sal, *P < 0.05 (one-way ANOVA followed by Holm-Sidak’s test). 0.3 Hz: *Fmr1*KO sal, *P < 0.05, WT VU, *P* > 0.05, *Fmr1*KO VU, *P* > 0.05, compared to WT sal. *Fmr1*KO VU compared to *Fmr1*KO sal, **P < 0.01 (one-way ANOVA followed by Holm-Sidak’s test). **e** Relation between VOR gain and fast phases frequency. 0.6 Hz: *Fmr1*KO sal, *P < 0.05, WT VU, *P* > 0.05, *Fmr1*KO VU, *P* > 0.05, compared to WT sal. *Fmr1*KO VU vs *Fmr1*KO sal, *P < 0.05 (Kruskal–Wallis followed by Dunn’s test). 0.3 Hz: *Fmr1*KO sal, **P < 0.01, WT VU, *P* > 0.05, *Fmr1*KO VU, *P* > 0.05, compared to WT sal. *Fmr1*KO VU versus *Fmr1*KO sal, *P < 0.05 (Kruskal–Wallis followed by Dunn’s test). **f** Sociability test set-up. **g** Discrimination Index between mouse-containing and empty cages is measured and compared to the WT sal (n = 31): *Fmr1*KO sal (n = 30, **P = 0.0047); WT VU (n = 31, *P* = 0.8410); (*Fmr1*KO VU (n = 32, *P* = 0.9321). (**P = 0.0064, *Fmr1*KO VU compared to *Fmr1*KO WT sal). **h** Social novelty test set-up. **i** Discrimination Index between unfamiliar and familiar mice is measured and compared to WT sal (n = 31): *Fmr1* KO sal (n = 30, ***P < 0.0004); WT VU (n = 31, *P* = 0.2219); *Fmr1* KO VU (n = 32, *P* > 0.1959). (*P = 0.0185, *Fmr1*KO VU compared to *Fmr1*KO WT sal). Two-way ANOVA followed by LSD’s test (**g, i**). Bar graphs show raw data and the mean. n is the number of mice used

The VOR helps to stabilize gaze when the head turns (Fig. 7A). The cerebellum plays an important role in the control of phase and gain dynamics of the VOR [17]. Illustrative examples of eye movements during table rotation at different frequencies are shown (Fig. 7A). As already described [37, 39], in control mice gain increases and phase angle decreases in the range of head frequency rotation (from 0.1 Hz to 0.6 Hz) used here. *Fmr1*KO sal mice showed significant deficits in gain (0.1 Hz: Kruskal–Wallis followed by Dunn’s multiple comparison test, H(3) = 12.53, *P < 0.05 compared with WT sal, Fig. 7B) and phase (0.6 Hz: one-way repeated measures ANOVA followed by Holm-Sidak’s multiple test, F(3, 41) = 3.58, **P < 0.01, Fig. 7C). VU 0155041 administration compensated the differences in gain (0.1 Hz: *P* > 0.05 compared to WT sal) (Fig. 7B), and phase (0.6 Hz: *P* > 0.05, Fig. 7C). In addition, *Fmr1*KO sal mice presented an increase in the number of fast phases per vestibuloocular cycle [one-way repeated measures ANOVA followed by Holm-Sidak’s multiple comparison test, F(3,41) = 8.03; 0.6 Hz: *P < 0.05; F(3, 41) = 6.62, 0.3 Hz: *P < 0.05 compared to WT sal]. Obviously, an excessive number of fast phases during the VOR would difficult an adequate vision, but this excess was compensated following VU 0155041 administration (0.6 Hz: *P* > 0.05; 0.3 Hz: *P* > 0.05, Fig. 7D*,* compared to WT sal; and 0.6 Hz: *P* < 0.05, 0.3 Hz: *P* < 0.01, Fig. 7D compared to *Fmr1*KO sal). The relation between vestibuloocular gain and fast phases frequency was also different between WT sal and *Fmr1*KO sal mice (Kruskal–Wallis followed by Dunn’s multiple comparison test, H(3) = 10.44, 0.6 Hz: *P* < 0.05; H(3) = 14.29 0.3 Hz: *P* < 0.01, Fig. 7E), and these differences were restored after VU 0155041 administration to *Fmr1*KO (0.6 Hz: *P* > 0.05, 0.3 Hz: *P* > 0.05, Fig. 7E compared to WT sal; and 0.6 Hz: *P* < 0.05, 0.3 Hz: *P* < 0.05, Fig. 7E compared to *Fmr1*KO sal). Then, VU 0155041 may offer some therapeutic relief to the motor learning deficits in FXS.

The cerebellum also controls some non-motor activities such as social cognition [58, 59]. Since social interaction deficits are a hallmark of the *Fmr1*KO mice [60, 61], we assessed whether VU 0155041 also ameliorates these impairments. The sociability test evaluates the time animals interact with a cage containing another mouse as opposed to that with an empty cage (Fig. 7F). For habituation each mouse was placed in the cage for 5 min with both boxes empty. During this phase the total distance travelled was similar in both genotypes (Two-way ANOVA followed by Bonferroni’s multiple comparisons test (F(3,114) = 29.27): P > 0.9999). However, VU0155041 increased this parameter in both phenotypes (***P < 0.0006, *Fmr1*KO VU compared to *Fmr1*KO sal, and **P < 0.0028, WT VU compared to WT sal, Additional file 4: Fig. S4).

*Fmr1*KO sal mice show a deficit in sociability compared to WT sal mice (two way ANOVA followed by LSD’s multiple comparison test, F(3,120) = 4.45, **P = 0.0047), whereas the treatment with VU01550 reverses this deficit (**P = 0.0064, *Fmr1*KO VU compared to *Fmr1*KO sal, Fig. 7G). Next, we evaluated social novelty by measuring the interaction time with a familiar as opposed to an unfamiliar mouse (Fig. 7H). *Fmr1*KO sal mice show a deficit in social novelty compared to WT sal mice (two-way ANOVA followed by LSD’s multiple comparison test, F(3,120) = 6.54, ***P = 0.0004), whereas the treatment with VU01550 reverses this deficit (*P = 0.0185, *Fmr1*KO VU compared to *Fmr1*KO sal, Fig. 7I). These results indicate that VU 0155041 ameliorates the alterations in social behavior of *Fmr1*KO mice.

## Discussion

In this study, an increase in basal synaptic transmission and the lack of presynaptic PF-PC LTP in *Fmr1*KO mice is shown to be a consequence of the increase in SV docking and priming, and in the RRP size, which precludes further potentiation of neurotransmitter release. These parameters can be restored by either diminishing the [Ca^2+^]_e_ concentration or through a decrease in Ca^2+^ influx at PFs induced by the mGluR4 PAM, VU 0155041. *Fmr1*KO mice also show deficits in skilled reaching, classical eyeblink conditioning and in the VOR. Interestingly, administration of VU 0155041 ameliorates these motor learning deficits and the impaired social interactions of these *Fmr1*KO mice.

The RRP of SVs is made up of docked and fully primed SVs, whose membrane fusion can be triggered by Ca^2+^ influx. The number of docked SVs is strongly related to the size of the RRP [14, 15] and, therefore, an increase in tightly docked and fully primed SVs may explain the increased aEPSC frequency at *Fmr1*KO synapses. However, it is important to understand why *Fmr1* KO synapses have more docked/primed SVs in order to design strategies to revert this phenotype. Two protein interactions influence the docking and priming of SVs: the formation of the Munc13-Rim1-Rab3 complex [62]; and the assembly of the SNARE complex promoted by the priming activity of Munc13 [63]. As such, neurons deficient in Munc13s have no docked SVs [41] and Munc13-2 deficient synapses fail to display the mGluR7 receptor mediated potentiation associated to enhanced SV docking/priming [64]. Munc13-1 is translocated to membranes upon DAG binding [65, 66], promoting its interaction with RIM1 to form a heterodimer with priming activity [67]. In addition to the DAG binding C1 domain [68, 69], Munc13-1 is also activated by Ca^2+^ through its Ca^2+^-CaM [70–72] and Ca^2+^-phospholipid binding domains [73]. Cerebellar granule cells express Munc13-1 and Munc13-3, the latter also containing binding sites for Ca^2+^ and DAG [74]. It is likely that the increase in Ca^2+^ and DAG of *Fmr1*KO synapses is responsible for the increase in SV docking and RRP size and the occlusion of presynaptic potentiation including the loss of PF-PC LTP. Then, the lack of PF-PC LTP is the result of the absence of dynamic range to enhance SV docking as synaptic vesicles are already docked under basal conditions. It is also likely that the decrease in [Ca^2+^]_e_ dampens Munc13 activity and that this provokes a reduction in the RRP size of sufficient magnitude to counterbalance the effect that elevated Ca^2+^ and DAG levels may have in *Fmr1* KO synapses [12, 75].

Reducing [Ca^2+^]_e_ from 2.5 to 1 mM, re-establishes β-AR mediated potentiation and PF-PC LTP at *Fmr1*KO synapses. However, as this strategy has limited translational potential, we tested whether PF-PC LTP can be also rescued pharmacologically by activating presynaptic responses that reduce Ca^2+^ influx at PF synaptic boutons. We found that mGluR4 activation, either by the group III mGluR agonist L-AP4 or the mGluR4 specific PAM VU 0155041, restores PF-PC LTP. This response is consistent with the presynaptic localization of these receptors at PF-PC synapses [76], and with their capacity to depress synaptic transmission and presynaptic Ca^2+^ influx [50]. VU 0155041 also restores the Ca^2+^ responses of cerebellar synaptosomes to those of WT synaptosomes.

We found that the acquisition of motor skills that affect movements involved in reaching and grasping is impaired in *Fmr1*KO mice as shown previously by [20], consistent with the deficient fine motor skills acquisition of FXS patients [16]. The motor skills required for reaching and grasping are high-level functions that involve multiple circuits and brain regions, including the cerebellum, basal ganglia and motor cortex [77]. The cerebellum contributes substantially to the coordination of skilled movements, such as those involved in reaching to grasp tasks [53, 54]. Thus, patients with lesions in the cerebellar cortex have impaired reach to grasp movements [78]. At the cellular level, PCs encode limb movements during reaching tasks [53]. It is well established that classical eyeblink conditioning and VOR represent two forms of cerebellar motor learning. For many years the prevailing view has been that cerebellar motor learning depends on postsynaptic LTD at the parallel fiber to Purkinje cell synapses [79]. However, it has also been shown that postsynaptic LTP also contributes to cerebellar motor learning providing a reversal of the LTD induced synaptic changes [19]. Now we found that cerebellar motor learning may also depend on presynaptic PF-PC LTP, thus widening the dynamic range of the synaptic changes that control this cerebellar function.

In addition to the lack of presynaptic PF-PC LTP described here, other changes at PF-PC *Fmr1* KO synapses include spine elongation and enhanced postsynaptic LTD [56], both of which are associated with motor learning deficits [80]. It is therefore attractive to hypothesize that the presynaptic changes at *Fmr1*KO PF-PC synapses (enhanced SV docking, RRP size and aEPSC frequency) could represent a compensatory mechanism to counterbalance the postsynaptic changes (enlarged and immature spines, and enhanced LTD) [56]. Interestingly, presynaptic LTP is also abolished in another brain areas in *Fmr1*KO mice, such as the anterior cingulate cortex [81].

We also found that rescuing PF-PC LTP with the mGluR4 PAM, VU 015541 favors the social interaction of *Fmr1*KO mice. Recent work revealed that the cerebellum controls not only motor functions, but also social behavior [58, 59]. Early developmental damage to the cerebellum is associated with deficits in social contact in autism [82], and cerebellar abnormalities that affect the structure and function of PCs have been detected in mouse models of autism [83], linking cerebellar dysfunction with autistic behavior. FXS patients also suffer cerebellar alterations, with a reduced size and density of PCs [84].

## Limitations

We are aware that one limitation of the study is that activating mGluR4s via systemic administration of VU0155041 can have broad effects that are likely to affect many neurons and circuits across several brain regions. However, cerebellar granule cells have the strongest expression of mGluR4 in the brain [85] and therefore, it is likely to be in this region where the effects of VU 155041 would be most significant. In addition, in most of the tests used in this study VU0155041 was administered once and therefore, the question whether a chronic regime of administration would increase its therapeutical value remains open.

## Conclusions

Our findings provide a clear rationale to explain how a structural change of PF-PC synapses such as the increase in SV docking, affects synaptic function causing the loss of synaptic potentiation and PF-PC LTP in *Fmr1* KO mice. These changes may be responsible for the motor learning and social deficit of *Fmr1 KO* mice. Our data points to the cerebellum as a potential target for pharmacological intervention to ameliorate motor learning and social skills of FXS patients.

## Supplementary Information

**Additional file 1. Fig. S1.** Absence of isoproterenol-induced potentiation of glutamate release in Fmr1KO cerebellar synaptosomes, despite normal β-AR expression and cAMP generation. (**A**, **B**) Mean traces from WT (**A**) and Fmr1 KO (**B**) cerebellar synaptosomes showing spontaneous release of glutamate in the presence of the Na+ channel blocker tetrodotoxin (1 μM, TTx), and in the presence or absence (control) of isoproterenol (100 μM). (**C**) Diagram summarizing the isoproterenol effect on glutamate release in the aforementioned conditions from WT (n=8/3 synaptosomal preparations: ***P<0.001) and Fmr1 KO synaptosomes (n=7/3 preparations: P>0.05). Basal release from Fmr1 KO vs WT synaptosomes (#P<0.05). (**D**) Western blot analysis of β1-AR in the P2 crude synaptosomes fraction from WT and Fmr1 KO mice. (**E**) The data were normalized to the WT values (n=3/3 preparations (Fmr1 KO, n=3/3 preparations: P>0.05). (**F**, **G**) Quantification of β-AR expressing cerebellar synaptosomes. Immunofluorescence of WT (**F**) and Fmr1 KO (**G**) synaptosomes stained with antibodies against β1-AR and the vesicular marker synaptophysin. (**H**) Co-expression of β-AR/synaptophysin in WT (24.9 ±1.1%, n=16,804/63 fields/2 preparations) and in Fmr1 KO synaptosomes (26.7 ±0.9%, n=18,712/75 fields/2 preparations, P>0.05). Scale bar in **F** and **G**, 5 μm. (**I**) The effect of isoproterenol on the cAMP levels in WT (n=14/3 preparations) is similar (P>0.05) to that in Fmr1 KO synaptosomes (n=15/3 preparations). Bar graphs show raw data and the mean. Two-way ANOVA followed by Tukey test in **C**. Unpaired student´s t test in (**E**, **H**, **I**).

**Additional file 2. Fig. S2.** Unaltered inhibition of synaptic transmission by VU0155041 in Fmr1KO mice. Quantification of EPSC amplitude (mean of 6 consecutive EPSCs delivered at 0.05Hz) 5 min after addition of VU0155041 (100 mM) in WT (n=11 cells/ 11 slices/ 6 mice) and Fmr1KO mice (n=11 cells/ 11 slices/ 6 mice). P=0.506, t=0.68, d.f.=20, unpaired Student´s t test, inhibition in WT compared to Fmr1KO. Bar graphs show row data of EPSC inhibition (%) and the mean. n, is the number of determinations/slices.

**Additional file 3. Fig. S3.** Intraperitoneal injection of VU0155041 rescues PF-PC LTP in slices of Fmr1KO adult mice. (**A**) Response to a 10 Hz stimulation in slices from VU and saline injected Fmr1KO adult (≥3 months) mice. Scale bars: 200pA and 20 ms. (**B**) amplitude (mean of 6 consecutive EPSCs delivered at 0.05Hz) 30 min after stimulation (2) compared to the respective values before stimulation (1): VU (5 mg/Kg) injected Fmr1KO mice (unpaired Welch´s test, t(6)=4.799, **P=0.003, n=6 cells/6 slices/6 mice); saline injected Fmr1KO mice (unpaired Student´s t test, t(12)=0.4428, P=0.666, n=7 cells/7 slices/6 mice). Bar graphs show raw data and the mean.

**Additional file 4. Fig. S4.** Fmr1KO mice show no change in the total distance travelled compared to WT mice, but VU0155041 increases this parameter in both genotypes. The total distance travelled (m) was measured during the habituation phase prior to the sociability and social novelty phases in WT sal (n=31), Fmr1KO sal (n=30, P>0.9999); WT VU (n=31, **P<0.0028), (Fmr1KO VU (n=32, ***P<0.0001) compared to WT sal. Fmr1KO VU compared to Fmr1KO sal, (***P<0.0006). Two-way ANOVA followed by Bonferroni´s test. Bar graphs show raw data and the mean. n is the number of mice used.

## Data Availability

All data generated or analyzed during this study are included in this article and its supplementary information files. All raw data are also available from the corresponding author upon reasonable request.
