# Supplementary figures and images for: The activation of mGluR4 rescues parallel fiber synaptic transmission and LTP, motor learning and social behavior in a mouse model of Fragile X Syndrome
Source: Mol Autism. 2023 Apr 7;14:14. doi: 10.1186/s13229-023-00547-4 (PMC10082511; doi:10.1186/s13229-023-00547-4)

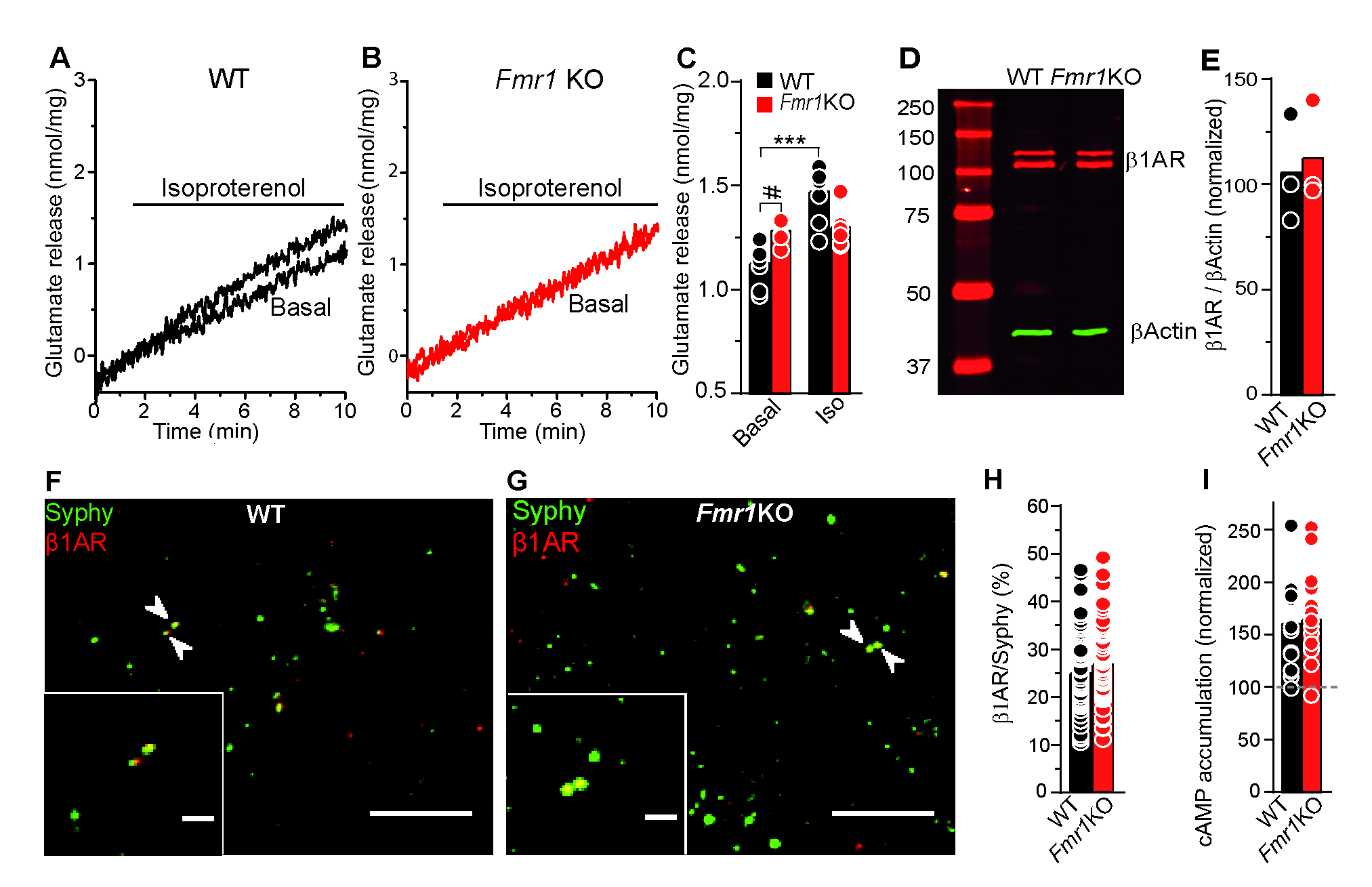

Supplement: Supplementary file 1 — Additional file 1. Fig. S1. Absence of isoproterenol-induced potentiation of glutamate release in Fmr1KO cerebellar synaptosomes, despite normal β-AR expression and cAMP generation. (A, B) Mean traces from WT (A) and Fmr1 KO (B) cerebellar synaptosomes showing spontaneous release of glutamate in the presence of the Na+ channel blocker tetrodotoxin (1 μM, TTx), and in the presence or absence (control) of isoproterenol (100 μM). (C) Diagram summarizing the isoproterenol effect on glutamate release in the aforementioned conditions from WT (n=8/3 synaptosomal preparations: ***P<0.001) and Fmr1 KO synaptosomes (n=7/3 preparations: P>0.05). Basal release from Fmr1 KO vs WT synaptosomes (#P<0.05). (D) Western blot analysis of β1-AR in the P2 crude synaptosomes fraction from WT and Fmr1 KO mice. (E) The data were normalized to the WT values (n=3/3 preparations (Fmr1 KO, n=3/3 preparations: P>0.05). (F, G) Quantification of β-AR expressing cerebellar synaptosomes. Immunofluorescence of WT (F) and Fmr1 KO (G) synaptosomes stained with antibodies against β1-AR and the vesicular marker synaptophysin. (H) Co-expression of β-AR/synaptophysin in WT (24.9 ±1.1%, n=16,804/63 fields/2 preparations) and in Fmr1 KO synaptosomes (26.7 ±0.9%, n=18,712/75 fields/2 preparations, P>0.05). Scale bar in F and G, 5 μm. (I) The effect of isoproterenol on the cAMP levels in WT (n=14/3 preparations) is similar (P>0.05) to that in Fmr1 KO synaptosomes (n=15/3 preparations). Bar graphs show raw data and the mean. Two-way ANOVA followed by Tukey test in C. Unpaired student´s t test in (E, H, I). [file 13229_2023_547_MOESM1_ESM.tif]

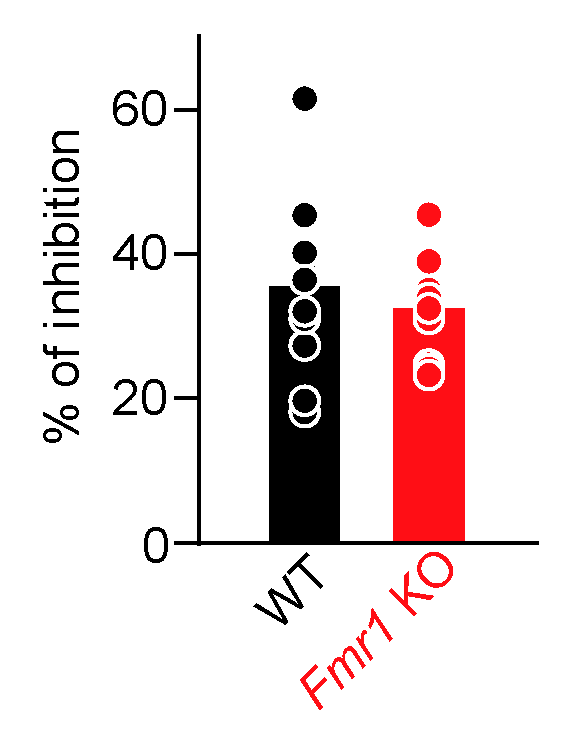

Supplement: Supplementary file 2 — Additional file 2. Fig. S2. Unaltered inhibition of synaptic transmission by VU0155041 in Fmr1KO mice. Quantification of EPSC amplitude (mean of 6 consecutive EPSCs delivered at 0.05Hz) 5 min after addition of VU0155041 (100 mM) in WT (n=11 cells/ 11 slices/ 6 mice) and Fmr1KO mice (n=11 cells/ 11 slices/ 6 mice). P=0.506, t=0.68, d.f.=20, unpaired Student´s t test, inhibition in WT compared to Fmr1KO. Bar graphs show row data of EPSC inhibition (%) and the mean. n, is the number of determinations/slices. [file 13229_2023_547_MOESM2_ESM.tif]

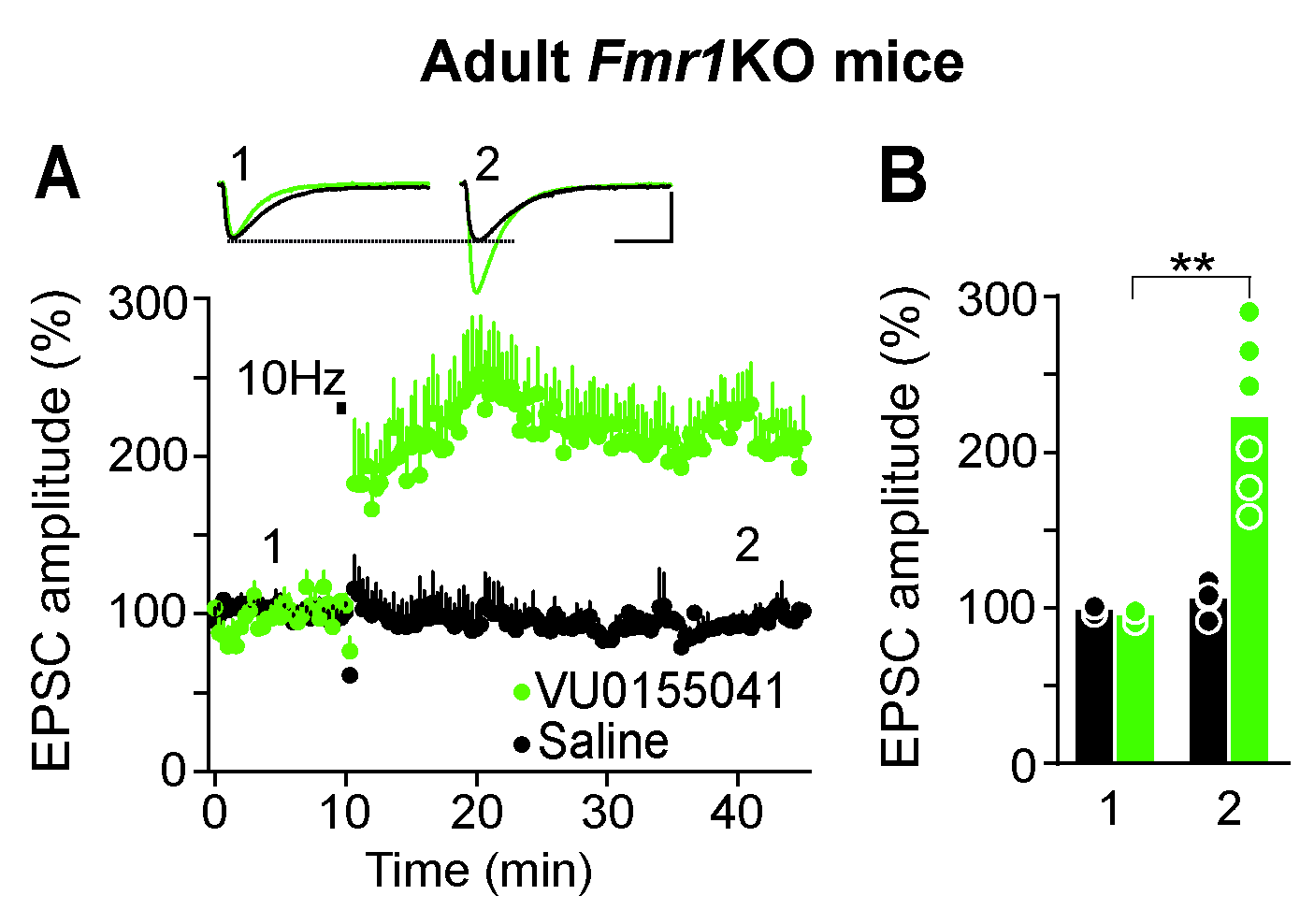

Supplement: Supplementary file 3 — Additional file 3. Fig. S3. Intraperitoneal injection of VU0155041 rescues PF-PC LTP in slices of Fmr1KO adult mice. (A) Response to a 10 Hz stimulation in slices from VU and saline injected Fmr1KO adult (≥3 months) mice. Scale bars: 200pA and 20 ms. (B) amplitude (mean of 6 consecutive EPSCs delivered at 0.05Hz) 30 min after stimulation (2) compared to the respective values before stimulation (1): VU (5 mg/Kg) injected Fmr1KO mice (unpaired Welch´s test, t(6)=4.799, **P=0.003, n=6 cells/6 slices/6 mice); saline injected Fmr1KO mice (unpaired Student´s t test, t(12)=0.4428, P=0.666, n=7 cells/7 slices/6 mice). Bar graphs show raw data and the mean. [file 13229_2023_547_MOESM3_ESM.tif]

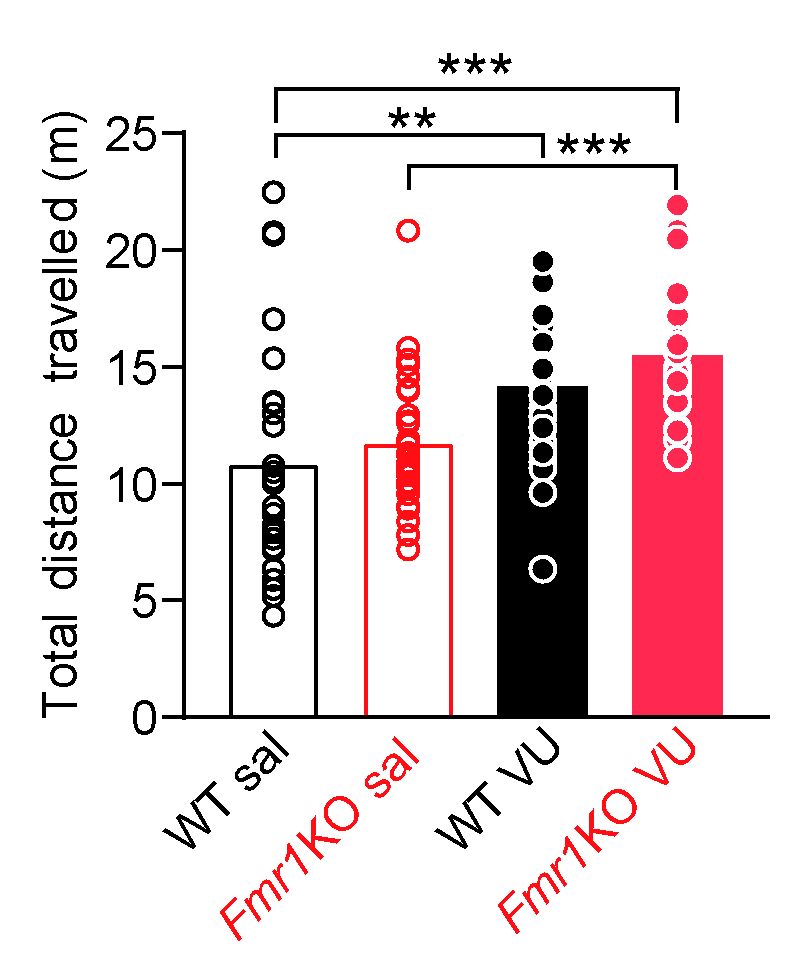

Supplement: Supplementary file 4 — Additional file 4. Fig. S4. Fmr1KO mice show no change in the total distance travelled compared to WT mice, but VU0155041 increases this parameter in both genotypes. The total distance travelled (m) was measured during the habituation phase prior to the sociability and social novelty phases in WT sal (n=31), Fmr1KO sal (n=30, P>0.9999); WT VU (n=31, **P<0.0028), (Fmr1KO VU (n=32, ***P<0.0001) compared to WT sal. Fmr1KO VU compared to Fmr1KO sal, (***P<0.0006). Two-way ANOVA followed by Bonferroni´s test. Bar graphs show raw data and the mean. n is the number of mice used. [file 13229_2023_547_MOESM4_ESM.tif]
